# Retrospective Evaluation of Livestock Monitoring Replay Controllers Under Limited Evidence and Distribution Shift

**DOI:** 10.3390/ani16213375

**Published:** 2026-10-26

**Authors:** Xiao Yang, Jinchang Zhang

**Affiliations:** 1 Department of Electrical Engineering & Computer Science, University of Wyoming, Laramie, WY 82071, USA; 2 Department of Computer Science, Indiana University, Bloomington, IN 47405, USA; zhanjinc@iu.edu

**Keywords:** precision livestock farming, evidence acquisition, dairy cattle monitoring, selective prediction, distribution shift, temporal memory, verification

## Abstract

Livestock monitoring systems must determine which records to inspect, assess whether the evidence supports an answer, and decide when to abstain. We constructed a retrospective replay benchmark using public MmCows records, with separate animals for development and evaluation. The benchmark included 120 current-state episodes and 62 scripted temporal and history checks from four evaluation cows. Across all 182 episodes, conditional routing used 2.51 stored-record calls per episode, compared with 2.82 for a fixed sequence. Task success was lower (90.11% versus 93.96%), while errors among accepted answers were higher (14/85 versus 2/68, or 16.47% versus 2.94%). Conditional routing did not save calls within the current-state episodes. Requiring two supporting cameras reduced errors and coverage, while the learned policy did not outperform a revisable fixed order. Raw confidence, temperature scaling, and isotonic calibration each retained 24 errors among 940 cases. Exploratory image analyses showed poor transfer to the Cow Behavior Video Dataset 5 (CBVD-5), and personalized forecasts showed no clear advantage over seven-day means. Verification accepted all 31 synthetically constructed correlated errors with valid record structure. The framework identifies limitations in evidence acquisition and verification, while the small number of animals and constructed task mixture limit conclusions about farm-level performance.

## Simple Summary

Livestock monitoring systems combine cameras, wearable sensors, and animal histories, but agreement between records does not guarantee a correct answer. We evaluated software that requests stored records, checks their compatibility, and either answers or abstains. The retrospective benchmark used records from four evaluation cows and deliberately included missing and conflicting evidence. Fewer record requests did not consistently improve the balance between correct answers and errors. Requiring support from two cameras reduced errors but also reduced the number of answered cases. Separate exploratory image tests showed poor transfer between datasets. Temporal and simulated-fault tests checked defined calculations and software rules; they did not validate animal health outcomes or real equipment-failure recovery. The contribution is a framework for examining these failure modes, rather than a new acquisition algorithm with superior performance. Prospective evaluation in additional animals and farms is needed before applying the findings to operational decisions.

## 1. Introduction

Livestock farms increasingly integrate cameras, wearable sensors, localization records, and environmental measurements to monitor animals over time [1,2]. These records inform decisions regarding whether an animal’s behavior or condition warrants attention. However, the data available at any decision point vary in completeness and reliability. Sources may be missing, conflicting, or based on stale historical context, and images may differ from the data used to train their models. The central challenge is determining when the available evidence is sufficiently reliable to support a farm management decision.

Research in livestock sensing has enhanced the individual tools that generate this evidence. Motion collars have been evaluated in 24 cows, and a noseband pressure system was validated for eating and rumination in 60 cows across 14 farms [3,4]. Visual models and sensor fusion extend the set of observable behaviors by combining complementary modalities [5,6]. These studies typically evaluate whether a sensor or model reproduces a reference standard under a defined data split. However, their metrics do not specify how a monitoring controller should act after tool outputs are produced, particularly when sources are unavailable, inconsistent, or interdependent.

A gap exists between producing measurements and deciding whether those measurements justify action. Although adding more sources may increase availability, it can also propagate upstream errors or introduce evidence collected for a different animal, time, or population. Consequently, the decision depends on the records already available, those that can still be obtained, and the cost of acquisition. Reference labels must remain excluded from this process to prevent confusing apparent support with correctness.

Evidence acquisition can be formulated as a sequential decision problem. After inspecting a record, a controller can request another source, consult animal history, or abstain if the available evidence is insufficient. Here, an agent means a stateful software controller that selects record-query and terminal actions under an access budget. The evaluated decision loop used deterministic rules or fitted acquisition values, without a generative language model. We therefore use replay controller for the implemented system. This operational definition distinguishes the present evaluation from language-model agents that generate plans or explanations.

We used MmCows to construct a retrospective replay benchmark [7]. Each episode specified a task contract, an access budget, an evidence ledger, and permitted animal history. Current-state action rules used camera records and sensor-model outputs rather than the manual behavior reference. A separate scoring step compared terminal outcomes with the reference and task contract. The integrated temporal routines instead replayed precomputed ankle-derived events and history-availability conditions. They are scripted implementation checks and do not provide an independent, reference-blind test of temporal reasoning.

The experiments examined missing or conflicting records, temporal reconstruction, visual distribution shift, and admissible fallback paths. The Cow Behavior Video Dataset 5 (CBVD-5) supplied a separate visual domain for crop-level analysis [8]. The visual classifiers were not synchronized with the replay controller. Results from later learned visual models were exploratory because CBVD-5 had already been inspected; they do not validate an end-to-end multimodal system.

The contribution is an evaluation framework and an empirical description of failure modes, not a superior evidence-acquisition algorithm. The framework records source requests and distinguishes correct answers, incorrect answers, appropriate abstentions, and other abstentions. Its main controller evidence comes from task-specific current-state comparisons; calibration, temporal reconstruction, retrieval, and synthetic fault tests assess separate components. The learned acquisition policy did not outperform its revisable fixed-order baseline. All population-level interpretations are limited by four evaluation cows and retrospective selection.

### 1.1. Livestock Sensing and Longitudinal Behavior

Livestock sensing studies provide examples of evaluations that separate animals across development and test datasets. Motion collars were evaluated in 24 cows [3], and a noseband pressure system was validated for eating and rumination in 60 cows across 14 farms [4]. Back-mounted inertial data have also supported unsupervised state modeling [9]. These studies compare behavior-specific sensors with reference methods that are more independent than endpoints reconstructed from the same signal.

Resource-aware accelerometer classification utilized approximately 19 annotated hours from ten cattle [10], whereas joint time and frequency learning employed millions of inertial samples from ten beef steers [11]. Accelerometry-GNSS fusion was evaluated using leave-one-animal-out designs across two datasets [6]. Our inquiry begins after these tools produce a record: how should a replay controller act when stored outputs disagree or become unavailable?

### 1.2. Public Cattle Data and Visual Transfer

Video-based cattle monitoring systems often leverage temporal context, which static crops omit. Hierarchical spatiotemporal models capture richer behavioral patterns [5]; architectures such as CNN-LSTM, C3D-ConvLSTM, and two-pathway models operate on clips or frame sequences [12,13,14]. A TimeSformer pipeline further integrates localization, tracking-assisted annotation, and manual agreement checks [15]. However, their reported accuracies are not directly comparable to the oracle crop experiment employed in this study.

MmCows provides synchronized records for replay experiments [7]. CBVD-5 contains concurrent posture and activity labels within some boxes [8]. Its release supplies no official training/test partition, although the publication separately reports a random 70/20/10 training/test/validation split for its demonstration model. Our source-only transfer analysis did not use that split. Cattle identity recognition addresses a separate objective [16]. Frozen ImageNet ResNet-18 features were included to examine representation sensitivity [17]. A review of 315 open livestock datasets identifies limitations in diversity and standardization [18], supporting careful reporting of label definitions and data partitions when interpreting transfer.

### 1.3. Agricultural Agents, Retrieval, and Tool Use

Agricultural language-model agents have largely been evaluated in advisory and knowledge-based tasks. NutriCHAT uses expert ratings, latency, and cost to evaluate poultry nutrition assistance [19], while CottonBot combines retrieval and tools for cotton management [20]. Retrieval augmented generation (RAG), ReAct, and Toolformer provide frameworks for retrieval, reasoning with actions, and tool use [21,22,23]. Our knowledge component used lexical retrieval followed by an answerability decision [24], without generated responses. A review of livestock housing agents distinguishes evidence for sensing and data access from the limited validation of autonomous barn-level control [25]. The present replay evaluation falls within this narrower evidence-acquisition scope.

### 1.4. Active Acquisition, Memory, and Repair

Active feature acquisition chooses the next costly observation from the information already available. Li and Oliva use a generative surrogate to provide uncertainty and information-gain signals to a sequential policy [26]. Efficient Dynamic Discovery of High-Value Information (EDDI) uses a partial variational autoencoder and expected information gain about target variables [27]. Janisch et al. formulate feature requests and classification as actions that trade classification error against acquisition cost [28]. Our fitted random-forest action values operate over stored record queries with explicit support and abstention rules. None of these general methods was implemented under the same states, budgets, and terminal rule, so the comparison is conceptual and does not establish algorithmic superiority. Animal memory and lineage checks extend the evaluation to temporal availability and permissible fallback paths.

### 1.5. Uncertainty, Shift, and Evaluation

Post hoc calibration and selective classification offer two mechanisms for controlling accepted errors. While temperature scaling performs effectively in source domains [29] and selective classification formalizes the risk-coverage tradeoff [30], deep ensembles provide more robust, albeit computationally expensive, uncertainty estimates [31]. However, these methods often degrade under distribution shift [32,33]. Furthermore, rare outcomes necessitate class-specific metrics and binomial confidence intervals [34,35].

Table 1 and Table 2 distinguish the technical scope of related studies from evaluation protocols that could be checked in accessible full texts. Table 2 is limited to those verified protocols; it is not a systematic review or a matched comparison of model performance.

## 2. Materials and Methods

### 2.1. Study Design, Data, and Reference Isolation

This study retrospectively analysed publicly available records from the MmCows and CBVD-5 datasets. No new animal experiments or farm data collection were conducted. Images and sensor measurements were obtained from the released datasets, and the original acquisition equipment and procedures are described in the corresponding dataset publications. The main analyses were conducted in Python 3.12.14 using NumPy 2.5.3, pandas 2.2.3, and scikit-learn 1.9.1. The analysis dependencies specified SciPy 1.18.1. Image processing for the handcrafted and pixel classifiers used Pillow 12.3.0, and figures were generated with Matplotlib 3.11.1. Frozen ResNet-18 features were extracted in a separate environment using PyTorch 1.9.0 and torchvision 0.10. The pretrained weights were identified by the filename resnet18-f37072fd.pth and a recorded SHA-256 checksum.

Cows C01–C03 were used for sensor cross-fitting and fusion, C04 for action-value fitting, C05 for calibration, C06 for threshold selection, and C07–C10 for evaluation. Sensor probabilities used to fit fusion on C01–C03 came from models excluding the corresponding cow. These partitions separate animals for fitting, but single-animal action, calibration, and threshold partitions provide little protection against animal-specific tuning. Later controller analyses reused inspected evaluation cows and are exploratory. Manual current-state labels were excluded from acquisition rules. Exported episode interfaces omitted references, but the integrated temporal implementation accessed precomputed event values; those cases are treated as scripted checks rather than independent blinded validation.

The integrated benchmark contained 182 unique animal–time episodes: four current-state families of 30 episodes, 30 temporal-change checks, and 32 history-insufficiency checks. All were clustered within C07–C10. The near-balanced family counts were chosen for stress testing, not to reproduce farm task prevalence. Current-state calibration used 940 cow–timestamps. Controller isolation retained 90 episodes, including 60 with a valid mandatory sensor and 30 without. Persistence and forecasting used 106 and 102 eligible origins, respectively. Visual evaluation included 686 held-out MmCows crops and 2531 mapped CBVD-5 crops. These crop and timestamp counts are not independent animal sample sizes.

Candidate current-state records were the first records in successive five-minute bins for each evaluation cow. Conflict candidates were those where the highest-ranked camera disagreed with the sensor-model argmax. Thirty were selected at evenly spaced positions in the sorted candidate list. From the remaining records, direct-observation candidates were drawn from every third entry, multimodal candidates from every second unused entry, and missing-sensor candidates from the remaining unused entries, again using evenly spaced selection. These four families used disjoint records. Temporal-change checks included both positive persistence cases and evenly spaced negative cases to reach 30; history-insufficiency checks used eight early origins per cow. The retained manifest contains 182 distinct animal–time pairs, but neighboring observations, shared scenes, and reused records across separate experiments remain dependent. Family counts were encoded before execution of these controller comparisons; selection still depended on source disagreement and precomputed temporal events, and was not random sampling.

For reporting, current-state controller outcomes are the principal empirical comparisons; fixed-predictor calibration is a secondary component analysis. Temporal reconstruction, forecasting, later controller extensions, learned visual transfer, and label-priority sensitivity are exploratory. Retrieval and declared-fault cases are software logic tests. This hierarchy describes the retrospective report and is not a claim of preregistered hypotheses or untouched confirmatory endpoints. Table 3 summarizes the evaluation populations, reference splits, and limits on independence.

### 2.2. Controller State, Acquisition, and Terminal Rules

**Figure 1:**
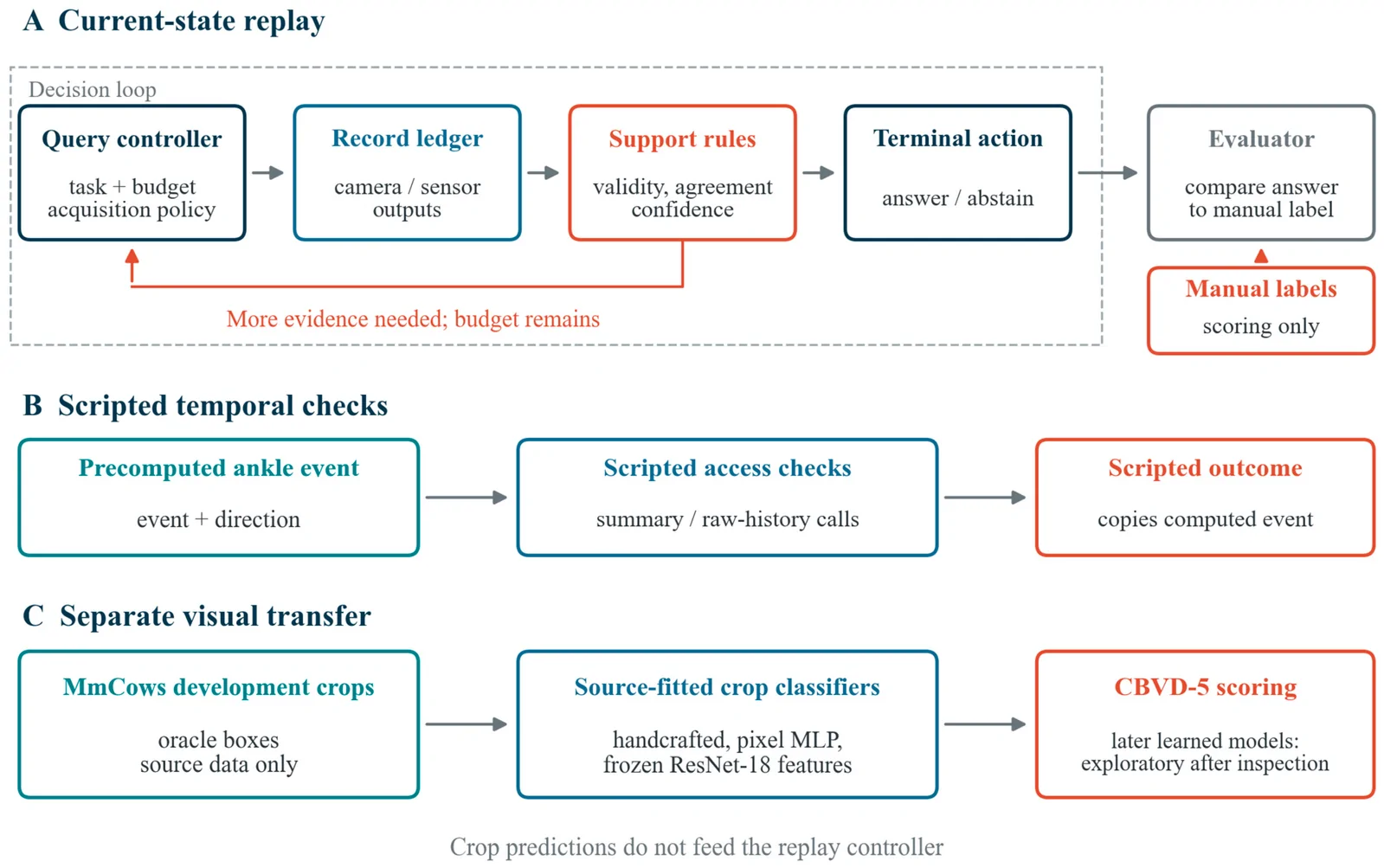
Scope of the retrospective evaluation. (**A**) The current-state decision loop uses acquired records while manual references enter the scorer. (**B**) Temporal routines use precomputed ankle events and are scripted checks. (**C**) The visual experiment evaluates source-fitted models separately; later learned-model results on CBVD-5 are exploratory.

At decision time t, the controller state was s = (q, E, M, b), comprising task contract q, acquired evidence ledger E, admissible history M, and remaining call budget b. A source was a record-producing stream or model; a tool was its query interface; a record was one returned item; evidence was the set of admissible acquired records; and a modality described the measurement type. Multiple cameras were separate sources within the visual modality. Query actions returned source, animal identity, requested and observed times, value or probability, availability, quality flags, and lineage. The terminal actions were answer and abstain. Structural support indicated that the required record pattern was present, not that the answer matched the hidden reference. Task success was a correct supported answer or an abstention required by the scoring contract. The tabulated evidence-sufficiency metric also credited correctly recognized insufficiency and therefore is not a pure source-agreement rate. Figure 1 distinguishes the current-state decision loop, scripted temporal checks, and separate visual transfer experiment.

### 2.3. Controller Comparisons and Calibration

The 182-case comparison evaluated complete controller designs, including their query limits and stopping behavior, rather than isolating acquisition order. Cameras were ranked by development accuracy as 4, 3, 2, 1. For direct observation, conditional routing queried at most the first three ranked cameras and stopped when two agreed; fixed routing always queried those three; all-context control queried all four cameras and the sensor. The terminal label was the most frequent available camera label, with ties resolved by first appearance in acquisition order. For multimodal, conflict, and missing-sensor tasks, conditional routing queried camera 4 and the sensor, abstained immediately if the sensor was unavailable, and otherwise queried cameras 3, 2, and 1 until one matched the fixed sensor candidate. Fixed routing always queried camera 4, the sensor, and camera 3; all-context control queried all five sources. These differences explain why additional evidence could support an incorrect sensor candidate.

For temporal fixtures, conditional routing used current and personal baseline summaries and added movement context for a precomputed positive event. Fixed routing always requested those three items; all-context control requested five. History-insufficiency checks used one, two, or five calls, respectively. The implementation read precomputed event values to form temporal answers and used the expected contract to credit abstention. These outcomes are therefore kept separate from reference-blind current-state inference.

The factorial experiment used the same 90 retained episodes to vary ranked order (4, 3, 2, 1) versus error-diversity order (3, 1, 4, 2), early versus exhaustive acquisition, and one versus two matching cameras. The mandatory sensor fixed the candidate. Five calls provided access to every source, and later disagreement could not revoke support; identical terminal outcomes across camera orders were therefore expected under full access. A separate three-call experiment made complete acquisition impossible. Two additional three-call controllers revised the candidate using the same fitted fusion model and calibration threshold, differing in fixed versus learned acquisition. Comparisons within this revisable pair isolate the tested ordering policy more closely; comparisons with fixed-candidate controllers remain descriptive because their terminal rules differ.

The revisable fusion model used 33 features. For each of five sources, requested and valid indicators were followed by three one-hot camera labels or sensor probabilities, with zeros for unavailable or unqueried values (25 features). Eight further features represented acquired-source and valid-source fractions, valid-camera fraction, sensor availability, presence of both modalities, largest camera vote fraction, number of distinct camera classes divided by three, and camera agreement with the sensor argmax. The fusion random forest used 150 trees, minimum leaf size 10, balanced-subsample weights, and seed 20260911. Each C01–C03 five-minute-bin record was expanded across all 31 nonempty source subsets; sensor probabilities for these animals were out of fold.

The action regressor used the 33 state features plus a five-element one-hot action vector. Its target was the change in fusion correctness after one query: I(ŷafter = y) − I(ŷbefore = y). It used 150 trees, minimum leaf size 12, and seed 20,260,912 on C04 states and permissible remaining actions. The selected action maximized predicted correctness gain minus 0.50 times the largest positive error correlation with an acquired source, plus 0.05 for introducing a new modality. Pairwise error correlations were estimated on C01–C04 where both sources were available; unavailable or constant-vector correlations were set to zero. Ties used source order camera 1, 2, 3, 4, sensor. Error-diversity camera ordering instead sorted signed camera–sensor correlations, yielding 3, 1, 4, 2.

Both revisable controllers began with camera 4. The fixed continuation was sensor, camera 3, camera 2, camera 1, truncated by the budget. After each query, the frozen fusion model could revise its argmax candidate. Acceptance required a valid acquired sensor and at least one camera agreeing with that candidate, together with isotonic confidence ≥0.90. Otherwise, querying continued until budget exhaustion or mandatory-sensor invalidity caused abstention. This controller calibration used 579 supported subset states from 62 C05 timestamps. Its threshold was chosen on C06 using the five-call controller and then reused at three calls. The C06 coverage achieved at that threshold was 61.29%, demonstrating that the nominal 90% target was not attained under the support constraints. This map is distinct from the 62-case fixed-two-source calibration comparison below.

The sensor tool was a 300-tree random forest (minimum leaf size 8; balanced-subsample class weights; seed 20260910), using speed, five-minute movement, ankle lying indicator, temperature, THI, and sine/cosine clock-time features [36]. The fixed-two-source calibration experiment froze the fusion prediction after camera 4 and the sensor had been acquired. Raw confidence was c = max_k_ p_k_, where p was the fusion class-probability vector. C05 supplied 62 first-per-20-min-bin cases for calibration; C06 supplied 62 for threshold selection; C07–C10 supplied 940 first-per-five-minute-bin cases for evaluation. Candidate labels and the two-query trace remained fixed.

Temperature scaling used q_k_(T) = exp(log(max(p_k_, 10^−10^))/T)/Σ_j_ exp(log(max(p_j_, 10^−10^))/T). Thus, log random-forest probabilities served as surrogate logits; this was probability-power scaling rather than access to native neural-network logits [29]. The scalar T minimized multiclass negative log likelihood on C05 using bounded optimization over [0.25, 8], yielding T = 1.0183938. Isotonic regression mapped raw maximum probability to z = I(ŷ = y), constrained to [0, 1] with out-of-range clipping. Each acceptance threshold was selected on C06 from 0 to 1 in steps of 0.005, minimizing distance from 90% coverage, then errors per all cases, then favoring the higher threshold. Thresholds were 0.765, 0.765, and 0.905 for raw, temperature-scaled, and isotonic confidence.

The binary Brier score was the mean of (c − z)^2^, where z indicated correctness of the fixed candidate; it was not a multiclass probability-vector score. ECE was Σᵦ(nᵦ/N)|mean(z)ᵦ − mean(c)ᵦ| over ten equal-width bins spanning [0, 1], with the final bin including 1 and empty bins contributing zero. Accepted-set scores were recomputed using only accepted cases. During revision, a post hoc sensitivity analysis exchanged C05 and C06 while retaining the frozen predictor. Per-animal reliability curves and metrics were also calculated. These checks do not replace nested animal-grouped refitting of the entire model pipeline.

### 2.4. Temporal Memory, Forecasting, and Visual Tools

The persistence endpoint was algorithmically defined from three consecutive two-hour ankle-derived lying fractions ending at the decision time. Each fraction was compared with the same clock interval over the preceding seven days. Eligibility required at least 80% of the expected minute records in each current window and at least five sufficiently observed baseline days. The development threshold was 0.2863095, the 75th percentile of absolute deviations on C01–C06. A positive event required all three deviations to be at least this threshold in the same direction. The reference, personalized summary, and raw-history calculations shared the ankle stream, and the raw-history and personalized branches assigned their predictions directly from the computed reference event and direction. Their agreement is therefore an implementation property, not independent reconstruction or biological validation. Forecasts targeted the subsequent six-hour ankle lying fraction and were compared with one-, three-, and seven-day clock-matched means. Clock matching partially accounts for recurring time-of-day patterns; it does not control breed, milk yield, temperature-humidity index, or other animal and environmental factors [37].

A post hoc definition sensitivity analysis retained the same 106 eligible evaluation origins and varied the development percentile (50th, 75th, or 90th), the number of latest consecutive two-hour windows (one, two, or three), and the same-sign requirement. Thresholds were estimated from 486 absolute segment deviations at 162 eligible C01–C06 origins. This analysis tested how the constructed event prevalence depended on the rule, without supplying an independent biological endpoint.

All visual models used oracle boxes with 5% padding and the same main three-class mapping. The handcrafted baseline used color, gradient, and box features. The pixel multilayer perceptron (MLP) used normalized 12 × 12 RGB inputs. The ResNet-18 model used official ImageNet-1K weights, a frozen 512-dimensional average-pooled representation, and a class-balanced logistic-regression head fitted on MmCows [17,38]. ResNet preprocessing resized the shorter image edge to 256 pixels, center-cropped 224 × 224 pixels, and applied ImageNet normalization. Source calibration used out-of-fold (OOF) probabilities from fits excluding one development cow at a time, with a development-only threshold targeting at least 80% pooled coverage. The encoder was not fine-tuned on MmCows or CBVD-5 labels. CBVD-5 labels were excluded from model fitting, but the target dataset had been inspected before the MLP and ResNet-18 analyses were specified; these learned-model transfer results are exploratory.

The visual partition differed from the controller partition. Four 15-min blocks were selected at fixed quantiles of the common camera timeline. C01–C06 in two blocks supplied 2256 source-fit crops; C05–C06 supplied 320 calibration crops; C07–C10 in a separate block supplied 686 test crops. The original calibration block contained only standing, so a nonoverlapping addendum was selected using label availability to include all three classes before its predictions were run. Learned visual models pooled the 2576 development crops for leave-one-cow-out prediction and final fitting. Development and test shared no animal IDs, frames, timestamps, or selected blocks, but used the same barn, day, and four cameras. Within development folds, different cows could share the same image background; those folds were animal-held-out rather than scene-held-out.

MmCows standing (ID 2), feed-up/feed-down (IDs 3/4), and lying (ID 7) were mapped to Standing, Feeding/foraging, and Lying. Unknown, walking, licking, and drinking were excluded; the selected source boxes contained 151 excluded cases (65 walking and 86 drinking). Feeding/foraging is an operational harmonization of dataset labels, not a demonstration that their definitions are biologically identical. CBVD-5 compact action IDs were 1 standing, 2 lying, 3 foraging, 4 drinking, and 5 rumination. Concurrent rows sharing video, time, box coordinates, and local entity ID were collapsed using lying > foraging > standing. Drinking and rumination remained in provenance but were not separate scored classes. All boxes with those labels also had a retained posture label.

The CBVD-5 compact test annotations contained 3761 action rows representing 2533 unique boxes. Two standing-plus-foraging boxes with invalid coordinates failed the minimum crop-size check and were excluded, leaving 2531 crops from 292 frames in 50 videos: 721 Standing, 789 Feeding/foraging, and 1021 Lying. Retained label combinations were standing only (650), standing plus lying (1), standing plus foraging (789), standing plus drinking (53), standing plus rumination (18), lying only (655), and lying plus rumination (365). Local entity IDs did not supply persistent animal identities across videos.

The MLP used standardized 432-dimensional RGB inputs, hidden layers of 64 and 16 ReLU units, Adam, L2 penalty 0.001, batch size 64, learning rate 0.001, and at most 80 iterations, with 15% internal early-stopping validation and patience 10. That internal split was row based. The ResNet-18 head used standardized features and balanced logistic regression (C = 1, lbfgs, maximum 2000 iterations). Model seeds were 20260911, incremented by held-out cow ID in OOF fits. The ResNet source threshold minimized accepted error subject to at least 80% OOF coverage, breaking ties by greater coverage and then the larger threshold on a 0.005 grid. The frozen weight file was resnet18-f37072fd.pth; extraction metadata recorded PyTorch 1.9.0.

**Figure 2:**
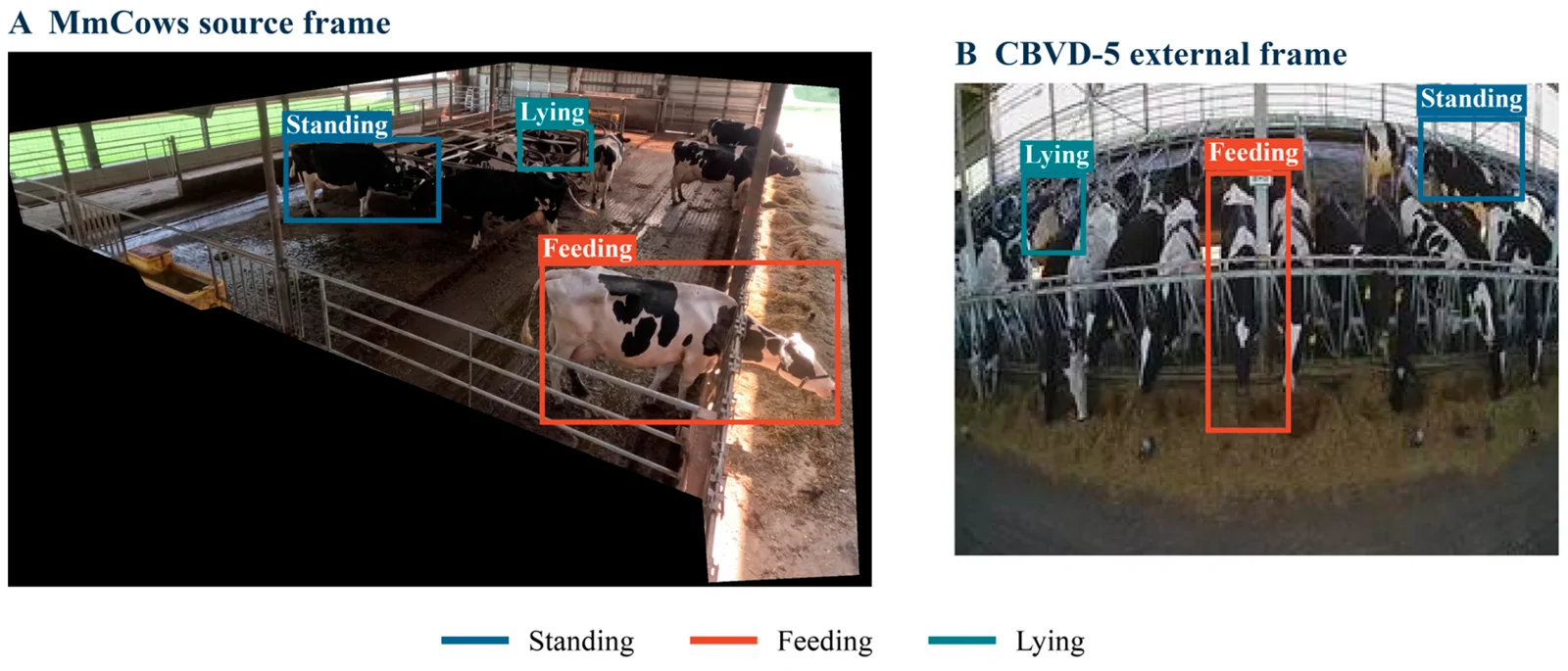
Illustrative largest-box examples from MmCows and CBVD-5. (**A**) MmCows source frame; (**B**) CBVD-5 external frame. The outlined boxes show the three-class mapping and differences in scene geometry, rather than a random sample of crop quality. Sources: MmCows [7] and CBVD-5 [8]. MmCows source-image terms are specified by its dataset release (currently CC BY-NC-SA 4.0); CBVD-5 version 11 was listed as CC0 in the archived download metadata. The top and bottom background margins of panel B were cropped to omit camera overlays; the selected animal boxes were retained.

Retained MmCows frames measured 4480 × 2800 pixels; CBVD-5 frames measured 256 × 256 pixels. MmCows center/width/height annotations and CBVD-5 corner annotations were converted to a common crop format. Figure 2 shows illustrative frames and the largest box for each mapped class. Largest-box selection can favor closer or less occluded animals and does not represent the typical crop distribution. The examples were selected independently of prediction correctness and were not additional training observations.

### 2.5. Grounding, Lineage, and Analysis

The retrieval corpus contained 12 fixed passages from dataset documentation and general knowledge sources, including temperature ranges [39], heat-stress guidance [40], lying-time context [41], and sensor-validation guidance [42]. The implementation contained 12 development questions (six answerable and six unsupported) and 28 evaluation questions (18 answerable and ten unsupported). Question–reference pairs were specified in the script before threshold fitting; reference passage IDs and answerability labels were used for scoring and withheld from the retrieval decision. Unsupported items asked for individual diagnoses, treatments, identities, costs, or other facts absent from the corpus. They were curated schema tests, not independently authored or adversarially validated questions. TF-IDF used word unigrams and bigrams with English stop words; BM25 used k1 = 1.5 and b = 0.75; keyword overlap used the fraction of unique query tokens present. Each threshold maximized exact supported decisions on development questions, with ties broken by answerability accuracy and then the higher threshold. Nine structured mutations separately tested identity, variable, time, unit, denominator, value, qualifier, evidence ID, and interpretation.

Lineage experiments were deterministic synthetic logic tests for model failure, derived-record corruption, invalid identity, and upstream ankle-device failure. Raw history was an admissible replacement only when identity and the upstream stream remained valid. These tests measured conformance to the specified gate, not naturally occurring failure detection or field recovery. Hierarchical bootstrap intervals used 2000 replicates: four animals were drawn with replacement, then each selected animal contributed its original number of calendar-day blocks sampled with replacement. Blocks were defined by floor (timestamp/86,400), and all paired episode differences in selected blocks were pooled for the mean. Percentile endpoints were the 2.5th and 97.5th percentiles. Each replicate could contain one to four distinct animals; resampling timestamps did not increase the four-animal sample size. These are descriptive resampling intervals, without an established nominal 95% coverage guarantee. Wilson intervals summarize rare-event counts under an independence assumption that repeated questions or animal observations may not satisfy. Crop-level metrics are descriptive because images and synchronized scenes are dependent.

For N episodes, task success was (correct supported answers + appropriate abstentions)/N. Coverage was accepted answers/N unless explicitly restricted to the 60 valid-sensor episodes. Accepted accuracy was correct answers/accepted answers; accepted-answer risk was incorrect answers/accepted answers. Errors per all episodes instead used N. We report counts with these denominators to avoid conflating low error incidence with reliable accepted answers.

Additional descriptive sensitivity enumerated all 4^4^ = 256 ordered animal-only bootstrap draws while retaining each animal’s complete case set, and omitted each evaluation animal in turn for paired forecast means. These checks kept fitted models fixed. Visual uncertainty used 2000 whole-cluster bootstrap replicates (seed 20261015): animals for MmCows and video IDs for CBVD-5, retaining all crops within a selected cluster. Macro-F1 used the fixed three-class set, including zero for absent classes; accepted accuracy was recalculated after the fixed threshold. These intervals condition on the fitted model and do not account for training or threshold-selection uncertainty. Target animal-level resampling was impossible because persistent identities were unavailable.

The archived preflight recorded Python 3.12.14, and retained package metadata identified NumPy 2.5.3, SciPy 1.18.1, scikit-learn 1.9.1, joblib 1.6.0, and Pillow 12.3.0. The original hierarchical random generator used seed 20260911. Stored scripts, model artifacts, case outputs, and selection manifests permitted the present methods and arithmetic audit; the absence of a persistent public code accession remains a reproducibility limitation.

Table 4 and Table 5 list the observed inputs, development choices, available state, terminal or reference rules, and reported metrics for each component.

### 2.6. Generative AI Assistance

OpenAI Codex (OpenAI, San Francisco, CA, USA) assisted code execution, audit construction, figure generation, manuscript drafting, and revision. The original chat archive recorded model identifier gpt-5.6-sol and Codex client version 0.151.0-alpha.7.2. Scripted integrity checks examined episode identifiers, trace counts, budgets, reference-export separation, prediction-row alignment, and document structure; the revision additionally compared methods against code and reproduced saved metrics. Such checks test computational consistency and do not constitute independent biological validation. The authors reviewed the outputs and remain responsible for the analysis and interpretation.

## 3. Results

### 3.1. Integrated Outcomes by Task Family

Across the 182 deliberately balanced replay cases, conditional routing, fixed sequence, and all-context control achieved task success of 90.11%, 93.96%, and 92.31%, with mean calls of 2.51, 2.82, and 5.00. Conditional routing returned 71 correct and 14 incorrect answers; fixed sequence returned 66 and 2; all-context control returned 75 and 14. Thus, errors among accepted answers were 14/85 = 16.47%, 2/68 = 2.94%, and 14/89 = 15.73%, respectively. Errors per all episodes were instead 14/182 = 7.69%, 2/182 = 1.10%, and 14/182 = 7.69%. The conditional-minus-fixed task-success difference was −3.85 percentage points, with a descriptive hierarchical 95% interval of −9.87 to −0.87 points; calls were lower by 0.319 (interval 0.205–0.565). These aggregate quantities include scripted temporal cases and reflect benchmark weighting, not expected farm-level performance.

Table 6 reports correct answers, incorrect answers, appropriate abstentions, and other abstentions as mutually exclusive outcomes. The 120 current-state episodes alone yielded task success of 85.00%, 90.83%, and 88.33% for conditional, fixed, and all-context controllers, with 3.017, 3.000, and 5.000 calls, respectively. Conditional routing therefore did not save calls in this subset. The lower overall call count arose from the scripted temporal families and missing-sensor checks. Per-family results should be interpreted before the aggregate summary.

Policy effects varied by task family. In direct observation, conditional and fixed routing each yielded 14 correct answers, 12 appropriate abstentions, and four other abstentions, resulting in 86.67% task success. All-context routing resolved the four additional abstentions to achieve 100% success but required 5.00 calls, compared with 2.67 or 3.00. In multimodal confirmation, conditional and all-context routing each returned 26 correct answers, three incorrect answers, and one appropriate abstention. Fixed routing produced one incorrect answer, three appropriate abstentions, and five other abstentions; success declined to 80.00% despite requiring 0.30 more calls than conditional routing.

Rankings shifted for conflicting evidence. The fixed sequence returned one correct answer, one incorrect answer, and 28 appropriate abstentions, achieving 96.67% task success. Conditional and all-context routing each returned one correct answer, 11 incorrect answers, and 18 appropriate abstentions, yielding 63.33% success. Although every policy satisfied the structural support rule in each conflict episode, structural support did not prevent incorrect answers.

**Figure 3:**
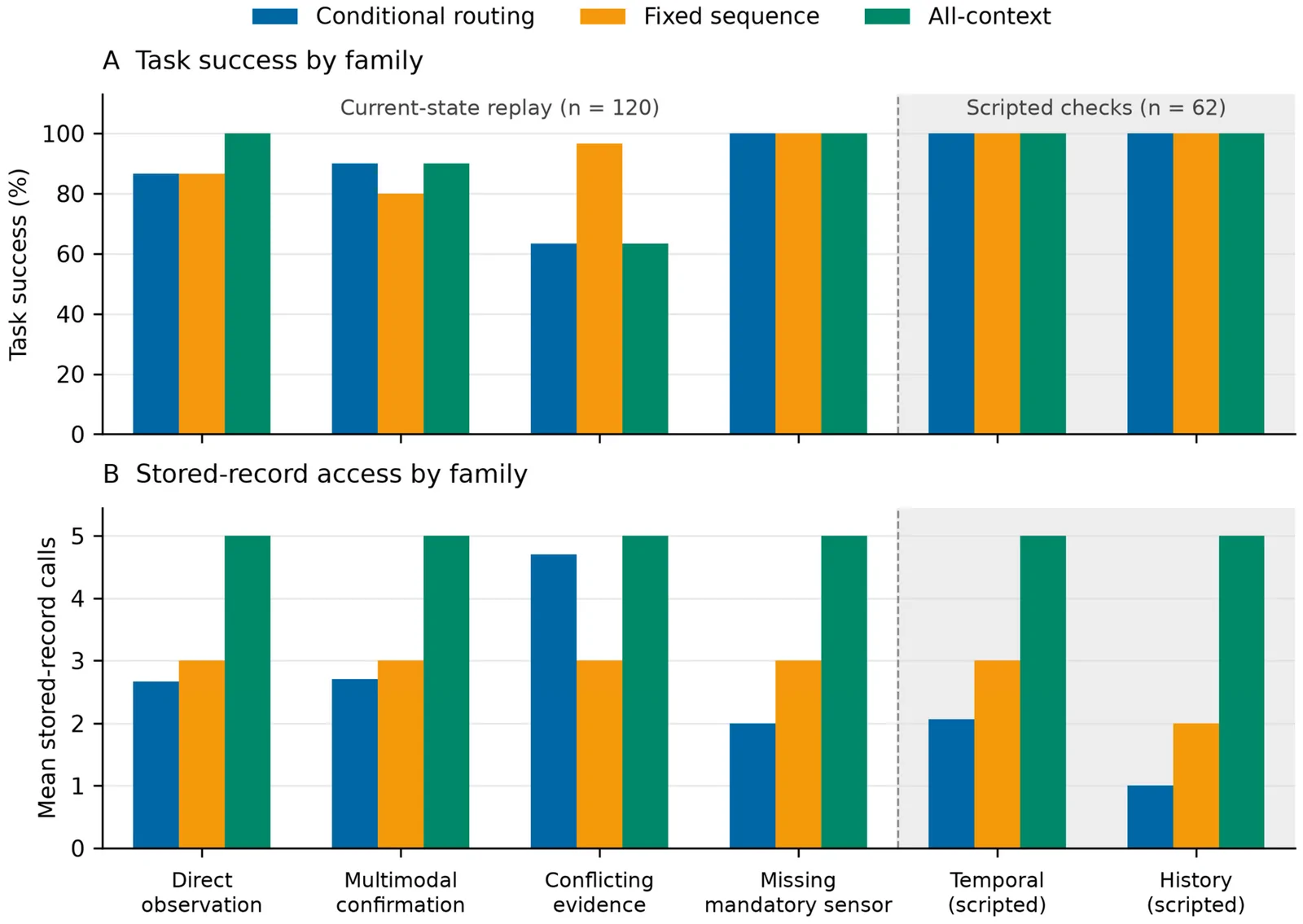
Task success and stored-record calls by task family. (**A**) Task success; (**B**) mean stored-record calls for each controller and task family. The first four families comprise 120 current-state episodes; the shaded final two families comprise 62 scripted temporal and history-insufficiency checks. Aggregate rates depend on this constructed task mixture.

All controllers matched the prescribed outcome for missing mandatory sensors, scripted temporal change, and scripted history insufficiency. Conditional routing used 2.00, 2.07, and 1.00 calls in these families; fixed sequence used 3.00, 3.00, and 2.00; all-context control used 5.00. The temporal branch read precomputed event values, so its perfect agreement and positive-event-dependent extra call are properties of the fixture. They are not evidence of independently acquired temporal reasoning or biological change detection. Figure 3 compares task success and mean record calls across the six families.

### 3.2. Rule-Dependent Acquisition and Calibration

Under the fixed candidate, full five-call access, and irrevocable-support rule, both camera orders reached the same terminal support set. This expected invariance is a design sanity check, not evidence that acquisition order is generally unimportant. Early stopping saved an average of 1.558 calls across factorial cells. Requiring two supporting cameras reduced incorrect answers from 14 to 2, reduced correct answers from 27 to 17, and increased abstentions from 49 to 71 among the same 90 episodes.

When one camera match was required, all four combinations of order and stopping criteria accepted 41 of the 60 episodes with a valid sensor. Coverage was 68.33%, accepted accuracy was 65.85%, and 14 of the 90 retained episodes resulted in incorrect answers. Requiring two camera matches accepted 19 of the 60 valid sensor episodes, yielding 31.67% coverage, 89.47% accepted accuracy, and only two incorrect answers. Early acquisition used 3.133 calls in ranked order and 3.144 calls in error-diversity order, whereas exhaustive acquisition used 5.000 calls. Since later disagreement could not withdraw support, the experiment measured how quickly a sufficient set was identified.

With a budget of three calls and one camera match, ranked acquisition yielded 22 correct and two incorrect answers. Acquisition ordered by error diversity yielded 19 correct and nine incorrect answers. Under the revisable fusion rule, fixed acquisition produced 21 correct and one incorrect answer, while learned acquisition produced 19 correct and one incorrect answer. The learned policy did not improve performance relative to the comparison at the same budget. All six policies abstained when the mandatory sensor was missing.

**Figure 4:**
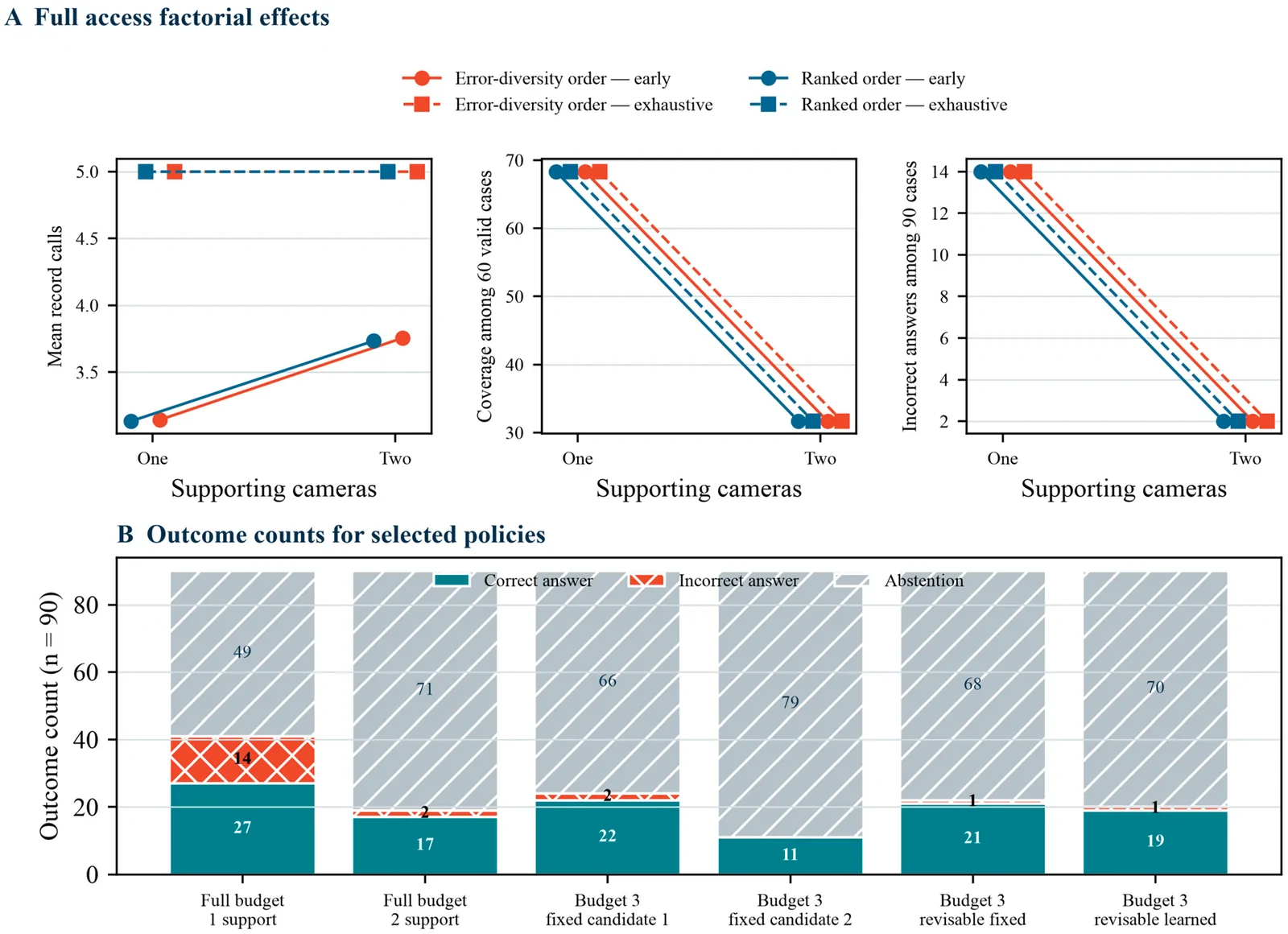
Controller effects and outcome counts. (**A**) All eight full-access factorial cells, showing mean record calls (left), coverage among 60 valid-sensor cases (middle), and incorrect answers among all 90 cases (right). (**B**) Correct answers, incorrect answers, and abstentions for selected full- and limited-access policies, with a common denominator of 90 episodes.

Among fixed candidate policies with limited access, requiring two camera matches eliminated accepted errors for both orders. Coverage among valid sensor episodes fell to 18.33% for ranked order and 11.67% for error-diversity order. With one camera match, error-diversity order covered 46.67% of valid sensor episodes, compared with 40.00% for ranked order, but it accepted nine errors instead of two. Revisable fixed and learned policies both used 2.456 calls. Their candidate revision rates were 4.44% and 2.22%, respectively, indicating that revisions were too rare to provide the learned order with an accuracy advantage. Figure 4 shows the full-access factorial effects and outcome counts for selected full- and limited-access policies.

Table 7 enumerates the eight full-access factorial cells. Coverage metrics utilize the 60 valid sensor episodes, whereas accepted errors and calls incorporate all 90 retained episodes, including the 30 episodes with an unavailable mandatory sensor. Table 8 reports controller outcomes under a budget of three calls.

Expected Calibration Error (ECE) summarized binned agreement between confidence and binary correctness; the binary Brier score summarized squared error for that same correctness event. On 940 fixed-predictor cases, raw confidence yielded ECE 0.0097 and Brier 0.0370, temperature scaling 0.0106 and 0.0370, and isotonic calibration 0.0242 and 0.0403. Each mapping accepted 24 incorrect answers at its development-selected threshold. Acceptance counts were 888, 886, and 887, respectively, so the acceptance decisions were not identical. No mapping reduced the number of accepted errors at these operating points. Table 9 reports calibration results across the development, threshold selection, and evaluation partitions.

Calibration and threshold selection each used only 62 cases from one cow. Isotonic calibration achieved ECE 0.0000 on its fitting cow C05, but 0.0220 on C06 and 0.0242 on pooled C07–C10 cases. Test coverage was 94.47%, 94.26%, and 94.36% for raw, temperature-scaled, and isotonic confidence, with accepted accuracy near 97.3%. These point estimates do not establish that a 90% development coverage target generalizes, nor that the small ECE difference between raw confidence and temperature scaling is meaningful.

Exchanging calibration and threshold-selection cows changed T from 1.0184 to 0.9074 and changed temperature and isotonic thresholds from 0.765 to 0.795 and from 0.905 to 0.875, respectively. Test coverages were then 94.47%, 94.36%, and 94.04%, with the same 24 accepted errors under each mapping. Raw ECE for C07–C10 was 0.0244, 0.0221, 0.0200, and 0.0187, exceeding the pooled 0.0097 in every animal. Exact animal-only bootstrap summaries gave descriptive ECE intervals of 0.0066–0.0210, 0.0082–0.0222, and 0.0159–0.0300 for raw, temperature-scaled, and isotonic confidence. These overlapping summaries do not support ranking methods by small pooled ECE differences.

**Figure 5:**
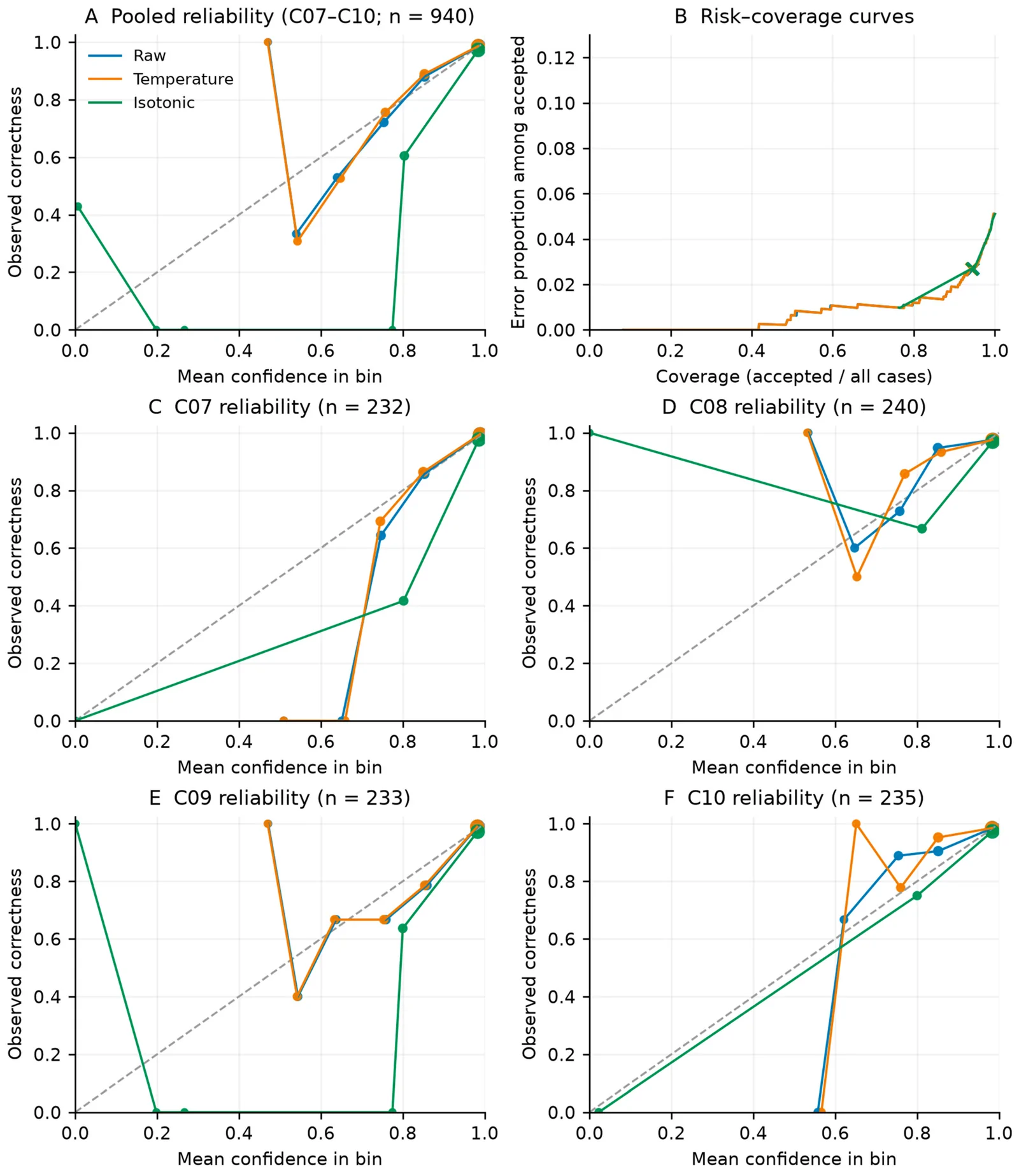
Pooled and per-animal calibration of the frozen two-source predictor. (**A**) Pooled reliability for C07–C10; (**B**) pooled risk–coverage curves; (**C**) C07 reliability; (**D**) C08 reliability; (**E**) C09 reliability; (**F**) C10 reliability. Reliability uses ten equal-width bins, with empty bins omitted; marker size reflects bin frequency. The dashed line indicates perfect calibration. Crosses on risk–coverage curves mark C06-selected thresholds. Per-animal curves are post hoc descriptive analyses.

Figure 5 shows similar risk–coverage behavior near the selected thresholds despite different reliability curves. The comparison fixes the predictor and two acquired sources, so it tests confidence remapping rather than acquisition. Calibration on one animal cannot establish reliability across animals, farms, or distribution shifts, and the separate 31-case synthetic correlated-error test is not a calibration evaluation on these 940 cases.

### 3.3. Exploratory Temporal Reconstruction and Forecasting

The persistence fixture contained 2 positive and 104 negative origins, with both positives from C09. The raw-history and personalized-summary branches directly assigned their outputs from the precomputed reference event and direction. Their 106/106 agreement is therefore an implementation property, not independently measured reconstruction accuracy or detection sensitivity. The always-no-change baseline achieved 98.11% accuracy but detected neither positive event; herd summaries also missed both positives and generated one false positive. Raw history exposed 2880 min records per episode, whereas summaries exposed aggregates, so equal call counts did not represent equal information volume.

**Figure 6:**
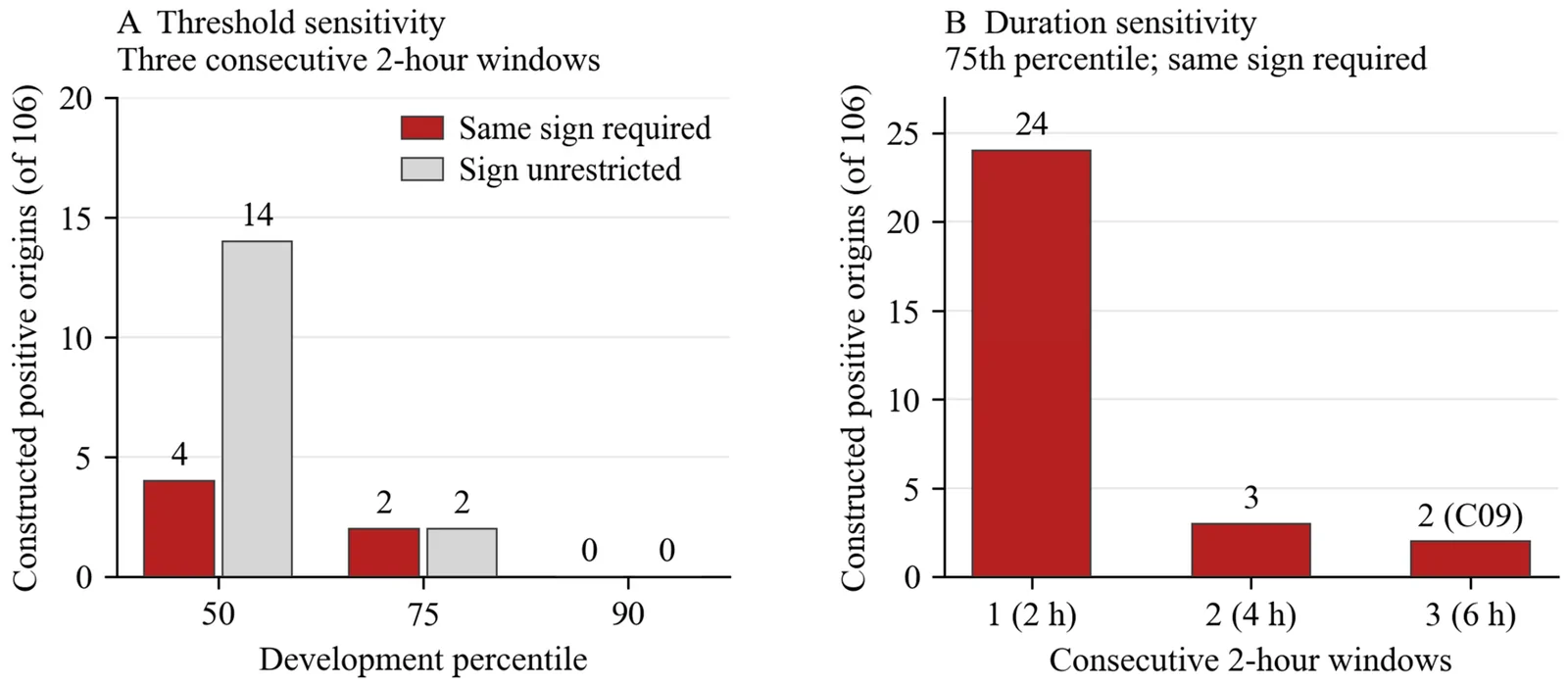
Post hoc sensitivity of constructed persistence-event counts among 106 fixed origins. (**A**) three consecutive two-hour windows across development percentiles, with and without a same-sign requirement. (**B**) one, two, or three latest two-hour windows at the original 75th-percentile threshold. Both positives under the original three-window rule belonged to C09.

Constructed event counts changed with the definition. With three consecutive two-hour windows, development thresholds at the 50th, 75th, and 90th percentiles produced 4, 2, and 0 same-sign positives among 106 origins; removing the sign requirement produced 14, 2, and 0. At the original 75th-percentile threshold, one, two, or three most recent windows with a common sign produced 24, 3, and 2 positives (Figure 6). These post hoc counts quantify definition sensitivity rather than clinical validity.

Across 102 common forecast origins, personalized ridge regression had a numerically lower MAE of 9.70 percentage points than the seven-day clock-matched mean at 10.43. The paired difference was −0.73 points, with a descriptive hierarchical 95% interval of −1.53 to 0.16 points. The interval includes zero and the evaluation involved only four cows, so the result does not demonstrate a clear forecasting advantage. Feature timestamps preceded the target six-hour interval, but this temporal ordering does not remove retrospective model-selection bias. Some development-animal fitting targets occurred later than evaluation origins, so this was not a prospective calendar-time holdout. Animal-only resampling gave an interval of −1.24 to −0.20 points, while omitting each evaluation animal gave paired means from −0.99 to −0.49. The change from a zero-crossing animal/day interval to a narrower animal-only interval shows sensitivity to the resampling hierarchy; it does not justify selecting the more favorable interval.

Results varied across animals. The personalized and seven-day MAE values were 7.10% and 7.00% for C07, 10.45% and 10.93% for C08, 12.60% and 14.05% for C09, and 8.44% and 9.49% for C10. Personalized memory reduced error for three animals but slightly increased it for C07. Although the average difference was small, effects varied among the animals, and the sample size was limited to four independent subjects.

**Figure 7:**
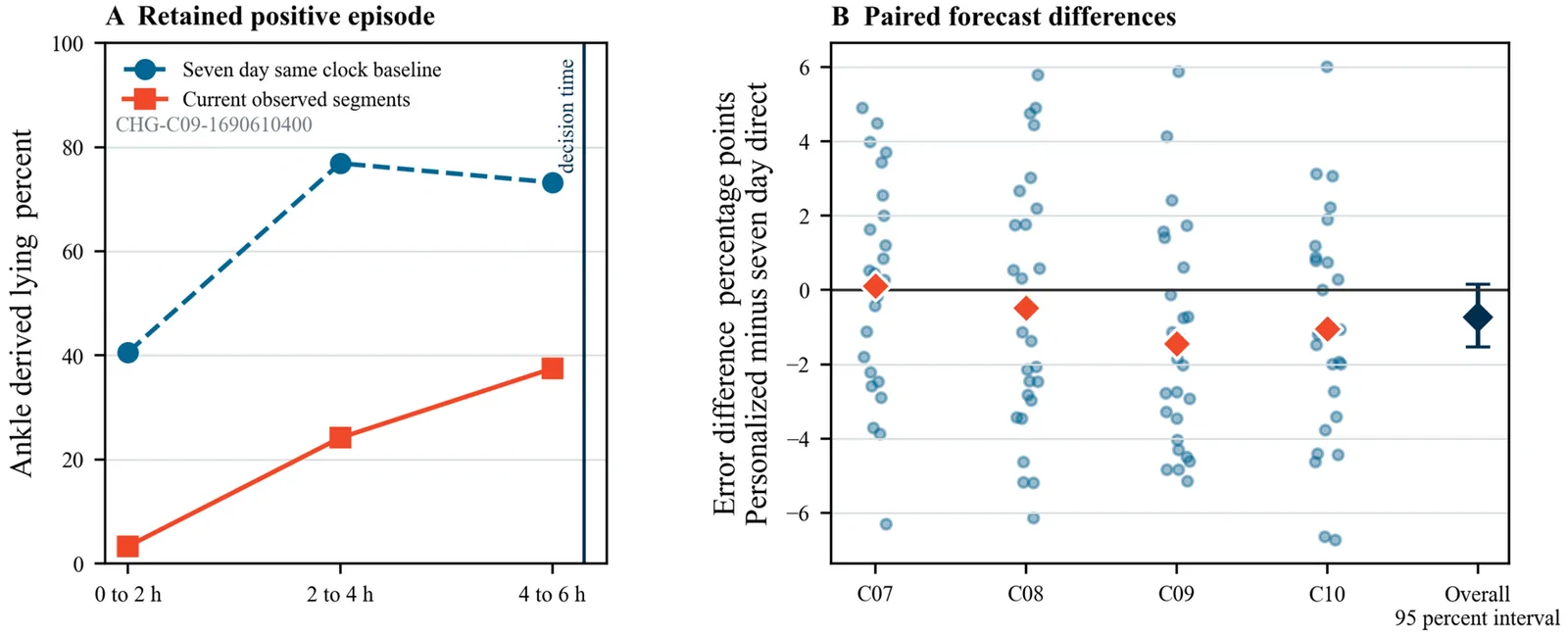
Animal-specific temporal evidence and paired forecast errors. Panel (**A**) displays the earliest retained positive persistence episode; Panel (**B**) compares personalized and direct seven-day forecast errors for all common cases. In panel (**B**), blue circles represent paired absolute-error differences at individual forecast origins, calculated as personalized ridge error minus the direct seven-day mean error. Orange diamonds denote the mean difference within each cow. The dark diamond denotes the pooled mean across all 102 origins, and its error bar is the descriptive 95% percentile interval from 2000 bootstrap replicates resampling animals and then calendar-day blocks within animals. Negative values favor personalized forecasts.

Figure 7 presents one retained persistence episode and the distribution of paired forecast errors. Panel A uses the earliest retained positive episode by identifier. Panel B includes every common forecast origin. The plotted deviation is calculated from sensor records and does not represent a diagnosed health or welfare event.

### 3.4. Exploratory Visual Transfer and Representation Sensitivity

On 686 held-out MmCows crops, the handcrafted baseline achieved 55.10% accuracy and 0.440 macro-F1; on 2531 CBVD-5 crops it achieved 27.42% accuracy and 0.148 macro-F1. The later pixel MLP achieved 51.02% and 0.312 on MmCows and 28.53% and 0.159 on CBVD-5. Frozen ResNet-18 features yielded 62.68% accuracy and 0.527 macro-F1 on MmCows, and exploratory CBVD-5 values of 29.04% and 0.167. Its CBVD-5 coverage was 94.55% and accepted accuracy 28.96%. The learned-model analyses followed target inspection and concern static oracle crops, not independent external validation of a monitoring system.

Post hoc cluster-bootstrap summaries for ResNet-18 gave MmCows accuracy 62.68% (descriptive interval 40.00–80.00%), macro-F1 0.527 (0.232–0.608), and accepted accuracy 65.20% (39.04–94.64%). CBVD-5 video-cluster intervals were 22.05–36.59% for accuracy, 0.134–0.199 for macro-F1, and 21.74–36.81% for accepted accuracy. All 60 source lying crops belonged to C07, and C10 test crops were all standing. These class–animal imbalances, four source animals, and unavailable persistent target identities sharply limit generalization from the intervals.

The improvement from ResNet varied by class. On held-out MmCows crops, the model correctly classified 370 of 421 standing, 17 of 205 feeding, and 43 of 60 lying crops. While standing and lying classification improved, feeding recall remained low. On CBVD-5, the model correctly classified 711 of 721 standing crops, 24 of 789 feeding crops, and none of 1021 lying crops. It assigned most feeding crops and all lying crops to the standing class.

**Figure 8:**
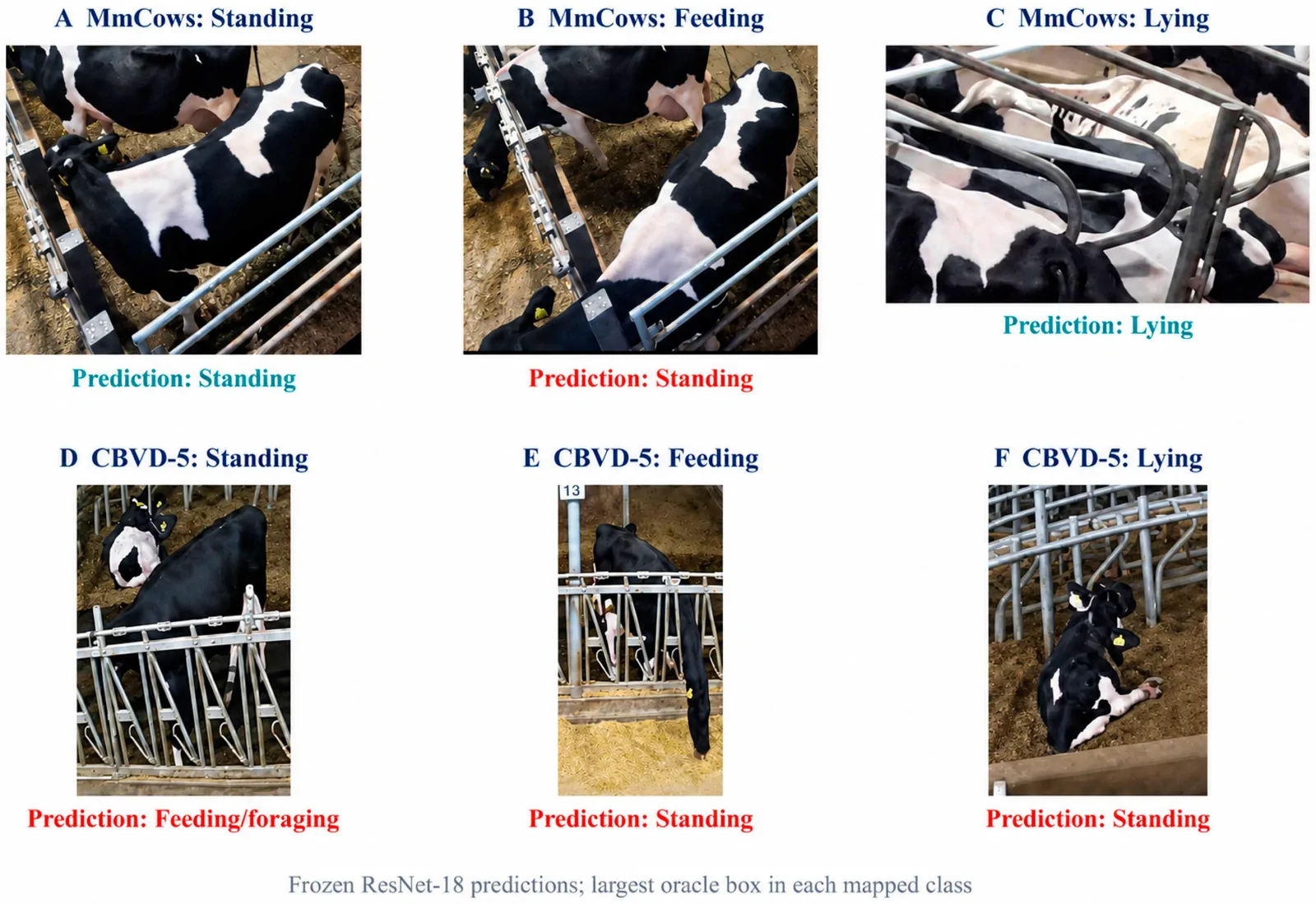
Illustrative largest-box crops and frozen ResNet-18 predictions from MmCows [7] and CBVD-5 [8]. (**A**) MmCows standing; (**B**) MmCows feeding; (**C**) MmCows lying; (**D**) CBVD-5 standing; (**E**) CBVD-5 feeding; (**F**) CBVD-5 lying. Headings give the mapped reference class, and labels beneath the images give the model prediction. Teal prediction labels indicate correct predictions and orange labels indicate incorrect predictions. Selection was independent of correctness and was not random sampling. MmCows source-image terms are specified by its dataset release (currently CC BY-NC-SA 4.0); CBVD-5 version 11 was listed as CC0 in the archived download metadata. The CBVD-5 examples retain the limited detail of the released 256 × 256 frames; their displayed crops contain 63 × 92, 67 × 127, and 38 × 54 source pixels, respectively. No image detail was reconstructed.

Figure 8 shows illustrative largest-box crops for each mapped class and partition with ResNet-18 predictions. Selection was independent of correctness but favors large boxes and cannot characterize the typical crop distribution. Performance is instead summarized by the complete class denominators and confusion counts.

**Figure 9:**
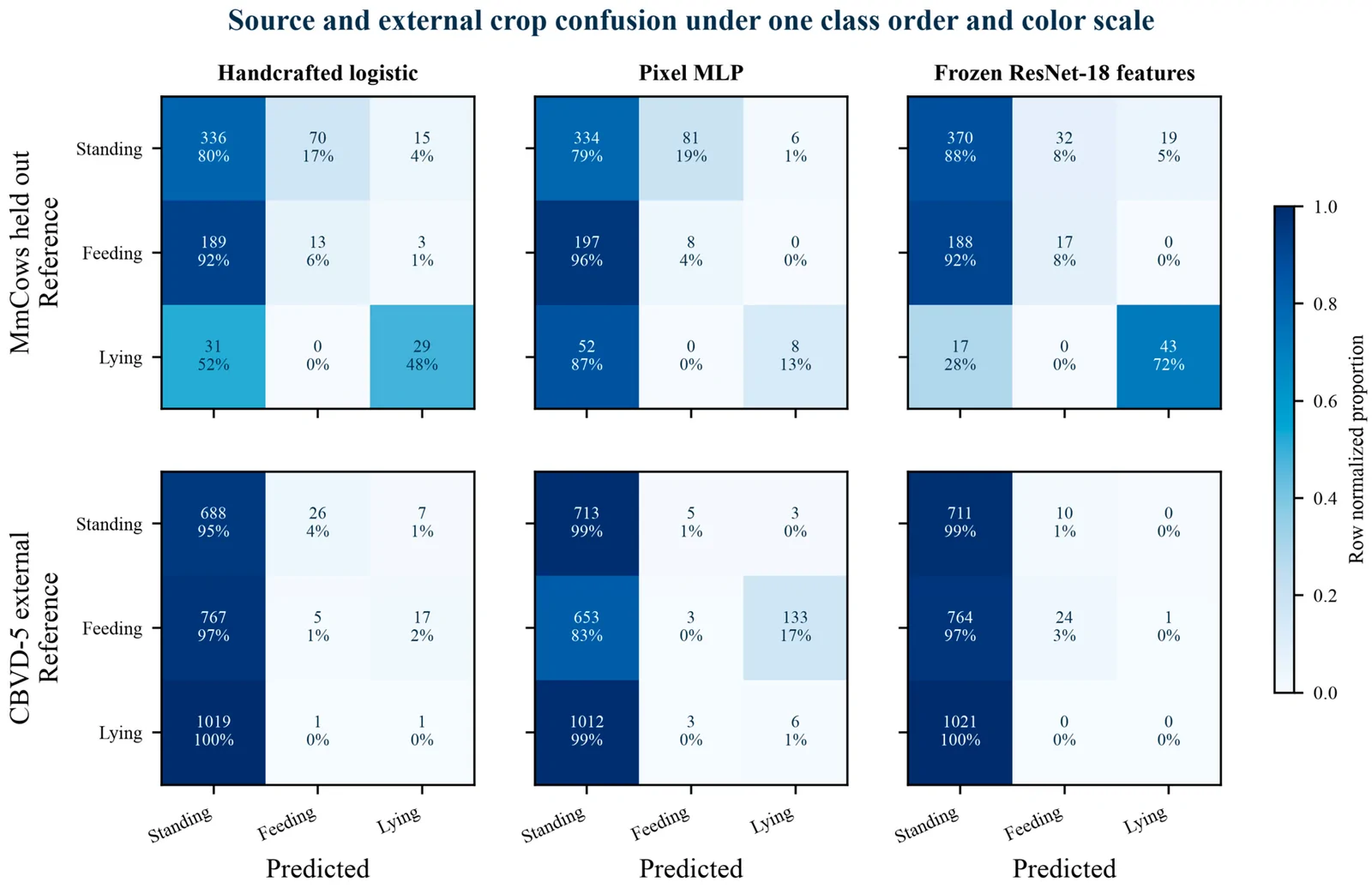
Confusion matrices for source and external oracle crops across three image representations. The upper-left, upper-middle, and upper-right panels show MmCows results for handcrafted logistic regression, the pixel MLP, and frozen ResNet-18 features, respectively. The lower-left, lower-middle, and lower-right panels show the corresponding CBVD-5 results. All panels share the same class order and row-normalized color scale; cells display counts and row percentages.

The target-majority oracle baseline used CBVD-5 labels to select the most common target class and achieved 40.34% external accuracy. It is a descriptive comparator unavailable in a label-free deployment. The source-majority constant predictor achieved 28.49%, and source-prior random prediction had expected accuracy of 31.56%, compared with 29.04% for ResNet-18. Table 10 summarizes the crop results and descriptive baselines. Figure 9 reports confusion matrices. The posture-only analyses change the reference task and are reported separately.

The frozen ImageNet-1K ResNet-18 encoder produced 5793 vectors of 512 features. It was not fine-tuned using MmCows or CBVD-5 labels; its generic pretrained weights were originally learned from labeled ImageNet images. Table 11 reports the performance of the frozen features and fitted linear head by partition.

Table 12 summarizes the exploratory sensitivity of CBVD-5 results to the reduction of concurrent labels.

Posture-priority reduction (lying > standing > foraging) reassigned all 789 standing-plus-foraging boxes from Feeding/foraging to Standing, leaving 1510 Standing and 1021 Lying cases and no feeding class. Its 58.28% accuracy therefore concerns a different two-class reference task. Restricting evaluation to standing-only or lying-only annotations retained 1305 crops and excluded 1226 concurrent-label crops; accuracy was 49.12% and macro-F1 0.329 over the two present classes. Neither analysis demonstrates improved three-class transfer. In the main task, 94.55% coverage coexisted with only 28.96% accepted accuracy.

### 3.5. Retrieval Grounding and Fault Lineage

TF-IDF and BM25 each retrieved the reference passage first for 17/18 answerable questions and within the top three for 18/18 (Table 13). Each achieved 23/28 exact supported decisions (82.14%; nominal Wilson 95% interval 64.4–92.1%). TF-IDF abstained on 8/10 unsupported questions and BM25 on 6/10. Keyword overlap abstained on all ten unsupported questions but answered only 7/28 questions. All nine structured claim mutations were detected. These are outcomes of a small curated schema test; the interval does not account for shared passages or question construction, and the results do not establish general retrieval or language-generation reliability.

In the deterministic lineage fixtures (Table 14), raw-history fallback reproduced all 60 lying-only answers after a declared model failure and all 60 after derived-record corruption. The gate prohibited fallback and abstained in all 60 identity-fault and all 60 upstream-device-fault cases. The 120 clean controls triggered no lineage fault flags. All 31 constructed, structurally valid correlated-error cases were accepted. These counts test the implemented logic under known fault declarations, not fault recognition or recovery rates under naturally occurring failures.

The clean controls yielded 98 supported answers and 22 ordinary abstentions, with no accepted errors. Their 81.67% answer rate does not reflect a lineage rejection rate. In contrast, all 31 correlated error cases remained structurally valid but were answered incorrectly. Provenance can determine whether an alternate record is admissible after a localized fault; however, it cannot establish the correctness of multiple admissible records.

## 4. Discussion

### 4.1. Acquisition Policy and Task Heterogeneity

The aggregate trade-off depended on the chosen task mixture. Conditional routing used 0.319 fewer calls per episode across all 182 cases, but this saving did not persist in the 120 current-state cases, where its mean call count slightly exceeded fixed routing. Conflict cases accounted for most extra errors: conditional and all-context controllers each accepted 11 incorrect answers versus one for fixed sequence. The descriptive bootstrap interval for the aggregate success difference excluded zero, but four animals and constructed task weights do not support a population-level superiority claim. Reports should separate current-state results from scripted checks and present error denominators explicitly.

All policies satisfied the structural support rule in all 30 conflict episodes. Nevertheless, conditional and all-context routing succeeded in 19 cases and failed in 11. While this rule confirmed the presence and support of required record types, it did not ensure agreement with the hidden reference. Shared environmental effects, synchronized artifacts, or a single upstream error can cause multiple sensor records to agree on an incorrect answer.

Full-access order invariance follows from a fixed sensor candidate, irrevocable support, and enough calls to reach all four cameras. It is specific to that terminal rule. With three calls, ranked order produced two errors and error-diversity order nine. Requiring two camera matches eliminated observed errors in those limited-access cells while accepting only 11.67–18.33% of valid-sensor cases. Zero observed errors under low coverage does not establish zero operational risk.

Revisable fusion allowed the candidate to change after each record. At the same mean call count, the learned order yielded fewer correct answers than revisable fixed acquisition, although both accepted one error. Candidates changed in only 2% to 4% of episodes, leaving few opportunities for the action model to alter the terminal result. While more general acquisition methods optimize explicit information objectives [26,27], the fitted action value policy tested here did not improve performance on this replay task.

### 4.2. Verification, Calibration, and Error Dependence

Requiring additional supporting cameras reduced both errors and coverage. Under full access, increasing support from one to two cameras decreased accepted errors from 14 to 2, while valid sensor coverage dropped from 68.33% to 31.67%. Under limited access, two-camera support eliminated observed errors but further reduced coverage. Although a workflow with human review may tolerate low automatic coverage, continuous autonomous monitoring necessitates a broader validation target.

Post-hoc calibration changes confidence rather than acquiring independent information. Positive-temperature scaling preserves each case’s predicted class [29]; a nondecreasing isotonic correctness map can merge distinct confidence values into ties. Neither can distinguish cases with identical calibration inputs. If correct and incorrect predictions occupy the same high-confidence region, thresholds chosen for approximately 90% development coverage may retain both. The 24 accepted errors under each mapping are consistent with this explanation, but do not prove that calibration can never reject correlated errors. Small single-animal fitting partitions and distribution shift can also impair calibration [32,33]. ECE depends on binning and the chosen calibration target [43], so its point estimates cannot by themselves establish which method is safer.

Lineage verification addressed a distinct problem. It enabled recovery from raw records following model or derived record failure, while blocking recovery when identity or the upstream device was invalid. However, it did not reject any apparently valid correlated errors across camera and sensor records. Provenance can verify an admissible evidence path, but it cannot verify the truth of several dependent sources. Correlated errors require independent evidence, direct tests of disagreement, or an external reference.

### 4.3. Temporal Memory and Biological Interpretation

The persistence tests evaluated access to an engineered ankle-derived event definition. Raw-history and personalized outputs copied the computed reference event, so perfect agreement did not establish independent calculation accuracy. Both positive origins came from one animal. Threshold, duration, and sign changes altered event counts, but no definition was validated against an independent welfare or health outcome.

The forecast target followed all input features, avoiding direct use of future target samples. Personalized ridge regression had a numerically lower MAE by 0.73 percentage points, but the descriptive interval crossed zero and only four evaluation cows contributed. Together with retrospective analysis choices, these limitations prevent a conclusion that learned memory improves forecasting in a new herd. Development-animal training targets also included calendar times later than some evaluation origins; animal separation should therefore not be confused with prospective time separation.

Further temporal evaluation requires animal- and farm-level separation [4,6,10], independently defined outcomes, more positive events, and a prospective time split. Sensitivity to event thresholds and window definitions should be reported separately from biological validity. Access reports should also distinguish information volume: 2880 raw minute records are not equivalent to one compact summary.

### 4.4. Visual Generalization and Dataset Geometry

Frozen ResNet-18 features achieved higher descriptive source accuracy than the handcrafted baseline (62.68% versus 55.10%) or pixel MLP (51.02%), but this did not establish reliable transfer to CBVD-5. Source gains were concentrated in standing and lying; feeding recall remained low. Dependence among crops and limited animal-level replication and unavailable persistent target identities preclude a strong inferential ranking of these representations.

On CBVD-5, accuracy was 29.04% and macro-F1 was 0.167. Figure 2 and Figure 8 illustrate differences between panoramic source scenes and compact target frames regarding resolution, camera geometry, cattle scale, background, and annotation composition. Confusion matrices indicate that the model assigned nearly all target crops to the standing class. Although the images provide context for this failure, they do not identify a single cause.

Source-based confidence did not yield reliable external predictions. Coverage on CBVD-5 was 94.55%, yet accepted accuracy was only 28.96%. Concurrent labels in CBVD-5 also introduce reference ambiguity. Prioritizing posture or retaining only unambiguous cases alters both the class set and the prediction task. These analyses help interpret the main three-class result but do not replace it.

The visual experiment classified oracle crops; it did not evaluate detection, tracking, identity, temporal behavior, sensor synchronization, or field latency. Consequently, its static crop results are not directly comparable to temporal video pipelines [13,15]. Integrating vision into the controller would require detection that preserves identity, time alignment with sensor records, and prospective evaluation of the complete controller.

### 4.5. Retrieval Grounding and Claim Checking

Retrieval rank and the final answer assessed distinct components of the system. Although TF-IDF and BM25 retrieved all answerable passages within the top three results, each method yielded only 23 exactly supported decisions out of 28 questions. Abstention on unsupported questions relied on a threshold derived from development data. While the keyword overlap method abstained on all unsupported questions, it answered only seven questions. Consequently, retrieval recall alone did not fully characterize the final decision.

The system successfully detected all nine structured claim mutations involving identity, variable, time, unit, denominator, value, qualifier, evidence ID, and interpretation. This evaluation was limited to the defined schema and excluded paraphrases, conflicting passages, cross-document synthesis, or generated language. Comparisons with this lexical method require further experiments on the generator and action policy for generative RAG and language model controllers [21,22,23].

### 4.6. Implications for Replay-Controller Evaluation

Reporting requirements vary by experiment type. Controller studies should report outcomes for each task family, accepted errors, abstention types, coverage, access budgets, and terminal rules. Temporal studies must include leakage checks, results for each animal, intervals for rare events, reference independence, and information volume. Visual studies should present representative source and target images, confusion by class, label mappings, and selective performance. Grounding and repair studies need to report factual correctness separately from lineage validity [44,45].

Each operational choice entails specific costs. Reducing call frequency is beneficial only if errors do not concentrate within critical task families. Additional support reduces errors but compromises coverage. High source accuracy is insufficient if external transfer fails. Fallback mechanisms are valid only when faults occur outside the fallback lineage. These conditions must be verified before deploying a replay controller for farm decisions.

## 5. Limitations and Required Evidence

The effective evaluation sample comprised four cows from one dataset, with repeated timestamps and deliberately constructed task families. C04, C05, and C06 each supplied a single-animal development partition for action values, calibration, and threshold selection. This design is vulnerable to animal-specific fitting and cannot substitute for nested animal-grouped validation. Later controller analyses reused inspected test cows, and later visual models followed CBVD-5 inspection. Their results are exploratory; there was no untouched second external dataset or prospective field deployment.

The current-state controller, scripted temporal routines, and visual crop models assess different components. In particular, the temporal routine used precomputed event references for its response and conditional extra query. It therefore cannot substantiate reference-blind temporal reasoning. The crop experiment omitted detection, tracking, persistent identity, synchronized sensor integration, and field latency. The final visual test separated animals and selected time blocks, but development OOF folds shared image backgrounds across cows and all source data came from the same barn, cameras, and day [46].

Only two persistence origins were positive, and reference and predictor shared ankle-data lineage. Forecasting used retrospective animal separation; synthetic faults tested declared conditions rather than real fault detection. All 31 plausible correlated-error fixtures were accepted. Bootstrap intervals with four animals describe sensitivity within these records and have no demonstrated population coverage. Stored-record calls do not measure latency, energy, monetary cost, human workload, or farm benefit. Uncertainty and class imbalance should therefore remain explicit when interpreting all component results. The post hoc sensitivity checks retained fitted models and evaluation records; they do not provide nested animal-grouped refitting or independent confirmation.

The study did not evaluate language-model decision controllers or obtain independent expert usefulness ratings. Source-image provenance also requires release-specific treatment. MmCows images came from its visual_data.zip release; the current dataset card specifies CC BY-NC-SA 4.0, while the original supplement used CC BY-NC 4.0 and the extraction manifest did not record a repository revision. Archived metadata for downloaded CBVD-5 version 11 identifies CC0: Public Domain, separately from the article’s CC BY 4.0 license. Figure 2 and Figure 8 reproduce attributed frames and crops with overlays; their source-image terms remain applicable.

## 6. Conclusions

This study contributes a retrospective framework for examining evidence acquisition, abstention, and verification failures. Under a fixed candidate and irrevocable support, full access made camera-order invariance expected; restricted access exposed order-dependent outcomes. The learned revisable policy did not outperform its fixed-order comparator. Calibration remapped confidence without reducing the number of accepted errors at the chosen thresholds, and lineage checks admitted structurally valid correlated-error fixtures. The temporal and visual analyses remain reconstruction tests or exploratory findings. Four evaluation cows, inspected target data, shared temporal references, and synthetic faults leave biological validity, external generalization, and operational reliability unresolved. The results support more explicit evaluation contracts and failure reporting, not deployment-readiness claims.

## Figures and Tables

**Table 1:** Literature comparison of task, modality, model, and temporal state.

Study	Task and Species	Modalities	Controller or Model	Temporal State
Smith et al. [3]	Five-behavior cattle classification	motion collar inertial data	one versus all classifiers	windowed sensor features
Zehner et al. [4]	eating/rumination measurement in dairy cows	noseband pressure	sensor event algorithm	behavior bouts
Arablouei et al. [10]	four cattle behaviors	accelerometry	resource-aware classifier	sensor windows
Arablouei et al. [6]	animal behavior classification	accelerometry + GNSS	multimodal sensor fusion	sensor windows
Fuentes et al. [5]	15 cattle behaviors	video	hierarchical spatiotemporal deep model	video sequence
Wu et al. [12]	basic dairy cow behaviors	video	CNN-LSTM	video clips
Qiao et al. [13]	calf/cow behavior classification	30-frame video clips	C3D-ConvLSTM	clip sequence
Bai et al. [14]	four dairy cow behaviors	video	X3DFast two-pathway network	fast/slow video pathways
Vu et al. [7]	multimodal dairy cow monitoring dataset	cameras, UWB, ankle, implantable records	dataset benchmarks; no unified agent claimed	two week records; one annotated vision day
Li et al. [8]	five cow visual behaviors/states	video	published video benchmarks	video segments
Cao et al. [15]	five beef cattle behaviors	video	YOLOv8 with labels assisted by ByteTrack; TimeSformer	4974 video clips
Mandiga et al. [19]	poultry nutrition QA	documents + four expert tools	ReAct-style LLM agent	dialog/tool trace, not animal history
Li and Oliva [26]	generic costly feature acquisition	benchmark features	generative surrogate model/MDP	sequential acquisition state
Ma et al. [27]	high-value feature discovery	tabular benchmark/healthcare variables	partial VAE + expected information gain	observed feature mask
Ovadia et al. [32]	uncertainty under distribution shift	image/tabular benchmark inputs	multiple uncertainty methods	not applicable
Present study	dairy cow replay tasks and visual crop classification	stored camera/sensor records, ankle history, lexical corpus, oracle RGB crops	Deterministic and fitted replay controllers; three separate crop classifiers	Animal-indexed seven-day summaries and raw history

**Table 2:** Comparison of verified evaluation protocols, acquisition designs, external evidence, and relevance.

Study	Acquisition or Verification	Evaluation Unit and Split	External Validation	Relevance
Zehner et al. [4]	Noseband classifications checked against direct observation	60 cows on 14 farms; one observation hour per cow	Multifarm measurement validation	independent measurement validation contrasts with circular reconstruction
Arablouei et al. [6]	Feature or posterior-probability fusion	Leave-one-animal-out validation in collar and ear-tag datasets	Two sensor datasets from one field experiment; no cross-farm transfer	Animal-held-out fusion context; no acquisition-policy comparison
Bai et al. [14]	Two-pathway video classification; no sequential record acquisition	Behavior-stratified random video split: 80% training and 20% validation; animal-disjoint separation not established	Internal video evaluation; no cross-dataset validation	Temporal video comparator; scores not directly comparable with static crops
Vu et al. [7]	dataset synchronization, not an acquisition controller	10 instrumented cows; repeated timestamps	not a cross herd agent test	data source for present replay benchmark
Li et al. [8]	Annotated video benchmark and SlowFast baseline	Release has no official partition; publication demonstration uses random 70/20/10 training/test/validation	Internal demonstration; no cross-dataset transfer evaluation	Source of CBVD-5 visual transfer data
Cao et al. [15]	YOLOv8/ByteTrack-assisted annotation; two manual annotators, κ = 0.95	Six cattle; 4974 behavior clips; video release has no prescribed split	Single collection setting; no cross-dataset validation	modern annotation/tracking/temporal pipeline omitted by oracle crops
Li and Oliva [26]	Generative-surrogate-assisted sequential policy	General classification and regression benchmarks	No livestock transfer evaluation	Requires a matched implementation for performance comparison
Ma et al. [27]	Partial VAE and expected information gain	benchmarks and two healthcare applications	no livestock validation	Principled information objective; not implemented on this replay task
Ovadia et al. [32]	confidence/calibration evaluation	controlled increasing shift	explicit shifted distribution evaluation	supports separating source calibration from external reliability
Present study	counted acquisition, cross modal support, confidence threshold, lineage gate	120 current-state cases and 62 scripted checks; four evaluation cows	Separate CBVD-5 crop analysis; learned models exploratory after inspection	Retrospective stress test; no prospective field or language-model evaluation

**Table 3:** Evaluation populations, reference splits, and independence limits.

Component	Unit	N	Reference and Split	Independence Limit
Integrated workflows	task episode	182	C07 through C10 records or temporal contract	Four cows; 62 scripted temporal checks; constructed task mix
Current state	cow timestamp	940	manual behavior label C07 through C10	stored camera labels
Controller isolation	retained episode	90	60 valid and 30 missing sensor	post hoc reuse of inspected cows
Persistence	animal origin	106	Ankle-derived event; outputs copied from computed event	Two positives, both C09; shared reference
Forecast	eligible origin	102	future 6 h ankle fraction	retrospective animal held out
Visual transfer	oracle crop	686 and 2531	MmCows held out and CBVD mapped	Repeated scenes; 50 target videos; no persistent target animal IDs
Retrieval	question	12 dev and 28 eval	passage ID or unsupported	small fixed corpus

**Table 4:** Observed inputs, development data, and fitted models used in each component.

Component	Observed Input	Development and Model
Current state sensor tool	speed_cm_s; movement_5min_cm; lying; temperature_C; THI; hour_sin; hour_cos	class-balanced random forest with 300 trees; predictions from fits that leave one cow out for C01 through C03 fusion; final C01 through C03 model applied to C04 through C10
Integrated mixed workflows	question; animal; decision time; admissible tools; history limit; remaining five call budget; acquired ledger	conditional rule; fixed sequence ranked on development data; all-context control
Fixed candidate controller isolation	mandatory sensor class plus acquired camera hard labels	Ranked order 4, 3, 2, 1; error-diversity order 3, 1, 4, 2; retrospective reuse of inspected evaluation cows
Revisable limited access controller	fusion state vector encoding requested and valid sources; class probabilities; agreement and modality indicators	150-tree fusion forest C01–C03; 150-tree action regressor C04; controller isotonic map C05; threshold C06
Persistence memory	three consecutive two-hour ankle lying fractions ending at decision; same clock intervals over preceding seven days	threshold 0.2863095 equals C01 through C06 75th percentile of absolute segment deviations; at least five prior days with 80 percent coverage
Six-hour forecast	past only recent personal; previous personal; global; herd; and same clock features by method	ridge models on C01 through C06; direct time matched controls; evaluation C07 through C10; retrospective animal held out
External visual crop tools	oracle RGB crop with 5% padding; resize and normalization specific to each model	Handcrafted logistic; 12 × 12 pixel MLP; frozen ResNet-18 with source-trained logistic head; grouped OOF calibration
Grounding and lineage	12 passages and development/evaluation questions; evidence IDs; source and upstream lineage metadata	TF-IDF; BM25; keyword ranker with development only answerability thresholds; deterministic structural claim and lineage checks

**Table 5:** Available states, terminal or reference rules, and reported metrics for each component.

Component	Available Actions or State	Terminal or Reference Rule	Reported Metrics
Current state sensor tool	camera_1 to camera_4 hard labels; sensor_model class probabilities	manual MmCows behavior label is scorer only	Accuracy; coverage; accepted accuracy; errors per accepted answer and per all cases; calls
Integrated mixed workflows	specific to each task current or temporal tool registry plus stop answer or stop abstain	Correct supported answer or contract-required abstention; temporal outputs are scripted fixtures	task family outcome counts; task success; evidence sufficiency; accepted errors; calls
Fixed candidate controller isolation	four cameras and sensor; full five call or limited three call budget	sensor class never changes; answer after one or two acquired cameras match; otherwise abstain	correct; incorrect; abstentions; valid sensor coverage; accepted accuracy; calls
Revisable limited access controller	fixed acquisition ranked on development data or learned action value specific to the task; maximum three record calls	Revisable fusion class; camera–sensor support and confidence ≥0.90 from development calibration	correct; incorrect; abstentions; candidate revisions; coverage; calls
Persistence memory	No-memory, raw-history, herd, personalized, and always-no-change fixtures	Three same-sign threshold deviations; raw/personalized outputs copy the computed reference	Constructed positive/negative counts; rule-definition sensitivity; access volume
Six-hour forecast	no animal specific features; recent; herd; personalized ridge; recent persistence; previous day; three day and seven-day means at the same clock time	future six-hour ankle lying fraction begins at decision and is scorer only	common case MAE; paired case specific differences; animal/day hierarchical interval
External visual crop tools	Source-only fitting; later learned models exploratory after target inspection	Main priority lying > foraging > standing; posture-only alternatives are different tasks	accuracy; balanced accuracy; macro-F1; class specific recall; confusion; coverage; accepted accuracy
Grounding and lineage	retrieve or abstain; raw fallback only when identity and upstream ankle stream remain valid	answerability labels and claim references remain hidden at evaluation; declared identity or device faults prohibit fallback	Top-k recall; supported decisions; unsupported abstention; declared-fault gate outcomes

**Table 6:** Outcomes for the 182 replay cases by task family and controller. Temporal-change and history-insufficiency families are scripted checks; percentages use the N in each row. Evidence sufficient includes correctly recognized insufficiency.

Task Family	Policy	N	Correct	Errors	Appropriate Abstain	Other Abstain	Task Success	Evidence Sufficient	Calls
Direct observation	Conditional routing	30	14	0	12	4	86.67%	86.67%	2.667
Direct observation	Fixed sequence	30	14	0	12	4	86.67%	86.67%	3.000
Direct observation	All-context	30	18	0	12	0	100.00%	100.00%	5.000
Multimodal confirmation	Conditional routing	30	26	3	1	0	90.00%	100.00%	2.700
Multimodal confirmation	Fixed sequence	30	21	1	3	5	80.00%	83.33%	3.000
Multimodal confirmation	All-context	30	26	3	1	0	90.00%	100.00%	5.000
Conflicting evidence	Conditional routing	30	1	11	18	0	63.33%	100.00%	4.700
Conflicting evidence	Fixed sequence	30	1	1	28	0	96.67%	100.00%	3.000
Conflicting evidence	All-context	30	1	11	18	0	63.33%	100.00%	5.000
Missing mandatory sensor	Conditional routing	30	0	0	30	0	100.00%	100.00%	2.000
Missing mandatory sensor	Fixed sequence	30	0	0	30	0	100.00%	100.00%	3.000
Missing mandatory sensor	All-context	30	0	0	30	0	100.00%	100.00%	5.000
Temporal change (scripted)	Conditional routing	30	30	0	0	0	100.00%	100.00%	2.067
Temporal change (scripted)	Fixed sequence	30	30	0	0	0	100.00%	100.00%	3.000
Temporal change (scripted)	All-context	30	30	0	0	0	100.00%	100.00%	5.000
History insufficiency (scripted)	Conditional routing	32	0	0	32	0	100.00%	100.00%	1.000
History insufficiency (scripted)	Fixed sequence	32	0	0	32	0	100.00%	100.00%	2.000
History insufficiency (scripted)	All-context	32	0	0	32	0	100.00%	100.00%	5.000

**Table 7:** Full-access results for the 2 × 2 × 2 controller experiment.

Camera Order	Stop	Support	Valid Coverage	Accepted Accuracy	Incorrect	Errors/90	Mean Calls	Missing Sensor Abstain
ranked order	early	1	68.33%	65.85%	14	15.56%	3.133	100.00%
ranked order	early	2	31.67%	89.47%	2	2.22%	3.733	100.00%
ranked order	exhaustive	1	68.33%	65.85%	14	15.56%	5.000	100.00%
ranked order	exhaustive	2	31.67%	89.47%	2	2.22%	5.000	100.00%
error-diversity order	early	1	68.33%	65.85%	14	15.56%	3.144	100.00%
error-diversity order	early	2	31.67%	89.47%	2	2.22%	3.756	100.00%
error-diversity order	exhaustive	1	68.33%	65.85%	14	15.56%	5.000	100.00%
error-diversity order	exhaustive	2	31.67%	89.47%	2	2.22%	5.000	100.00%

**Table 8:** Controller outcomes under a three-call budget. Coverage uses 60 valid-sensor episodes; outcome counts use all 90 episodes.

Policy	Family	Correct	Errors	Abstain	Valid Coverage	Accepted Accuracy	Calls	Revision
Fixed candidate ranked support 1	fixed candidate limited budget	22	2	66	40.00%	91.67%	2.456	0.00%
Fixed candidate ranked support 2	fixed candidate limited budget	11	0	79	18.33%	100.00%	2.667	0.00%
Fixed candidate error-diversity support 1	fixed candidate limited budget	19	9	62	46.67%	67.86%	2.489	0.00%
Fixed candidate error-diversity support 2	fixed candidate limited budget	7	0	83	11.67%	100.00%	2.667	0.00%
Revisable fixed order	revisable fusion limited budget	21	1	68	36.67%	95.45%	2.456	4.44%
Revisable learned acquisition	revisable fusion limited budget	19	1	70	33.33%	95.00%	2.456	2.22%

**Table 9:** Calibration results for model development, threshold selection, and fixed predictor evaluation.

Mapping	Split	N	Cutoff	ECE All	Brier All	Coverage	Accepted Accuracy	Accepted Errors	ECE Accepted	Brier Accepted
raw	calibration cow5	62	0.765	0.0275	0.0413	90.32%	98.21%	1	0.0205	0.0208
temperature	calibration cow5	62	0.765	0.0285	0.0415	88.71%	98.18%	1	0.0183	0.0204
isotonic	calibration cow5	62	0.905	0.0000	0.0277	90.32%	98.21%	1	0.0000	0.0164
raw	threshold cow6	62	0.765	0.0393	0.0600	90.32%	94.64%	3	0.0316	0.0491
temperature	threshold cow6	62	0.765	0.0413	0.0602	88.71%	94.55%	3	0.0279	0.0491
isotonic	threshold cow6	62	0.905	0.0220	0.0555	90.32%	94.64%	3	0.0231	0.0472
raw	test cows 7 through 10	940	0.765	0.0097	0.0370	94.47%	97.30%	24	0.0038	0.0245
temperature	test cows 7 through 10	940	0.765	0.0106	0.0370	94.26%	97.29%	24	0.0047	0.0245
isotonic	test cows 7 through 10	940	0.905	0.0242	0.0403	94.36%	97.29%	24	0.0112	0.0252

**Table 10:** Crop-level visual results and descriptive baselines. The target-majority oracle uses target labels. MLP and ResNet-18 transfer results are exploratory after target inspection; F1 denotes macro-F1.

Condition	MmCows Accuracy/ Macro-F1	CBVD-5 Accuracy/ Macro-F1	CBVD-5 Coverage	CBVD-5 Accepted Accuracy	Role
Handcrafted logistic	55.10% 0.440	27.42% 0.148	96.56%	27.41%	Initial frozen baseline
Pixel MLP	51.02% 0.312	28.53% 0.159	96.33%	29.16%	exploratory
Frozen ResNet-18	62.68% 0.527	29.04% 0.167	94.55%	28.96%	exploratory
Target-majority oracle	61.37% 0.254	40.34% 0.192	100%	same as accuracy	Uses target labels; descriptive only
Source majority	61.37% 0.254	28.49% 0.148	100%	same as accuracy	deployable constant
Source prior random	45.35% 0.310	31.56% 0.288	100%	same as accuracy	analytical expectation

**Table 11:** Frozen ResNet-18 feature and linear head performance by partition.

Partition	N	Accuracy	Balanced Accuracy	Macro-F1	Coverage	Accepted Accuracy	Accepted Errors	Calibrated ECE
MmCows grouped OOF development	2576	80.43%	59.70%	0.587	80.28%	85.93%	291	0.0652
MmCows held-out test	686	62.68%	55.95%	0.527	69.53%	65.20%	166	0.1562
CBVD-5 exploratory transfer	2531	29.04%	33.88%	0.167	94.55%	28.96%	1700	0.6262

**Table 12:** Exploratory sensitivity of CBVD-5 results to concurrent-label reduction. Feeding/foraging is a harmonized cross-dataset label, not proof of identical annotation definitions.

Mapping	N	Classes Present	Accuracy	Balanced Accuracy	Macro-F1 Over Present Classes	Interpretation
primary: lying > foraging > standing	2531	Standing|Feeding/foraging|Lying	29.04%	33.88%	0.167	Main mapping; learned-model analysis exploratory
posture priority: lying > standing > foraging	2531	Standing|Lying	58.28%	48.84%	0.368	789 feeding/foraging labels reassigned to standing
Unambiguous standing-only or lying-only labels	1305	Standing|Lying	49.12%	49.31%	0.329	1226 concurrent-label crops excluded; no feeding/foraging class

**Table 13:** Retrieval results following the application of the answerability threshold during development.

Ranker	Top 1 and Top 3 Recall	Answerability Accuracy	Unsupported Abstention	Exact Supported Decision	Coverage
TF-IDF	94.44% 100%	82.14%	80%	82.14%	60.71%
BM25	94.44% 100%	85.71%	60%	82.14%	78.57%
Keyword overlap	94.44% 100%	60.71%	100%	60.71%	25.00%

**Table 14:** Controlled synthetic lineage-logic tests. Accepted-error percentages use all cases in each row; no naturally occurring fault rate is estimated.

Condition	N	Fallback	Answer and Abstain	Errors/All Cases	Interpretation
Model failure	60	allowed	100% 0%	0%	local software fault
Derived record corruption	60	allowed	100% 0%	0%	raw stream bypass
Identity failure	60	prohibited	0% 100%	0%	animal assignment invalid
Upstream device failure	60	prohibited	0% 100%	0%	shared stream invalid
Clean controls	120	not needed	81.67% 18.33%	0%	No gate-triggered fault flag in declared clean cases
Apparently valid correlated error	31	not triggered	100% 0%	100%	Constructed verifier failure

## Data Availability

MmCows [7] and CBVD-5 [8] are available from their original data providers. The study archive retains analysis scripts, fixed configurations, model artifacts, case-level outputs, and dataset-selection manifests. A public persistent accession for this analysis archive is not provided. Public data are not redistributed here; the license and attribution requirements of the corresponding data release apply.
